# Role for Left Dorsomedial Prefrontal Cortex in Self-Generated, but not Externally Cued, Language Production

**DOI:** 10.1162/nol_a_00166

**Published:** 2025-06-12

**Authors:** Deborah Levy, Quinn Greicius, Catherine Wang, Edwin Ko, Duo Xu, John Andrews, Edward F. Chang

**Affiliations:** Department of Neurological Surgery, University of California, San Francisco, CA, USA; Graduate Program in Bioengineering, University of California, Berkeley, CA, USA; Department of Linguistics, Yale University, New Haven, CT, USA

**Keywords:** aphasia, dorsomedial prefrontal cortex, dynamic aphasia, neurosurgery, pre-supplementary motor area, transcortical motor aphasia

## Abstract

The left dorsomedial prefrontal cortex (dmPFC) is known to be associated with volition and motor function but is often overlooked in models of the neural bases of language. In this retrospective study, we reveal a robust statistical association between a rare language profile disproportionately affecting self-generated, but not externally cued, language production and damage to left dmPFC in a large (*n* = 307) neurosurgical database using both voxel-based and multivariate lesion-symptom mapping (VLSM, MLSM). This profile was not attributable to motivational or motor speech deficits. We further demonstrate that the probability of presenting with this profile is nearly 15 times higher following a resection in the dorsomedial prefrontal cortex than a resection elsewhere in the brain. Finally, we present a first person account of recovery from this language syndrome by a professionally trained linguist in the Supplementary Materials. These findings leverage a large dataset to add to the predominantly case-dominated literature demonstrating that damage specific to the dmPFC can cause a unique linguistic disturbance disproportionately affecting spontaneous speech, and provide a rare person-centered narrative of the experience of aphasia that is informative to scientists and clinicians alike. Overall, this work highlights the role of the left dmPFC, rarely included in dominant models of the neural bases of language, in the volitional control of fluent, self-generated speech.

## INTRODUCTION

For an aphasia-friendly version of this article, please see Supporting Information 1 of the Supplementary Data, available at https://doi.org/10.1162/nol_a_00166.

The pre-supplementary motor area (pre-SMA) is a region of cortex situated in the posterior third of the medial superior frontal gyrus, generally distinguished anatomically from the supplementary motor area (SMA) by the vertical plane passing through the anterior commissure (VCA line; Picard & Strick, 1996; Ruan et al., 2018). While the SMA proper projects directly to the primary motor cortex (PMC) and spinal cord and contains a somatotopic representation of the body relevant for movement, the pre-SMA projects instead to prefrontal cortex and is modulated by more abstract motor processes, such as action selection, motor learning, and/or preparation for complex movement (Eickhoff et al., 2011; Fontaine et al., 2002; Potgieser et al., 2014). Its precise functional role and boundaries remain a subject of extensive debate (Clairis & Lopez-Persem, 2023; Dadario et al., 2023; Nachev et al., 2008).

Cerebrovascular events in the territory of the anterior cerebral artery (ACA), the main arterial branch supplying medial frontal regions, are rare, accounting for less than 2% of all intracranial ischemic strokes (Mathew et al., 2018; Park et al., 2021). When they do occur, ACA strokes in the language-dominant hemisphere tend to be associated with transcortical motor aphasia, an aphasia classification broadly defined by impaired fluency with intact repetition (Crosson et al., 2018; Freedman et al., 1984; Sheppard & Sebastian, 2021). Deficits in motivation (i.e., abulia) and motor deficits are also often observed following infarcts to this region (Brugger et al., 2015; Hertrich et al., 2016; Park et al., 2021). However, it is rare for ACA strokes to damage the pre-SMA without damaging the SMA proper and vice versa, rendering it difficult to disentangle lesion-symptom relationships between the two (Binder et al., 2009; Mathew et al., 2018).

Neurodegeneration in the medial frontal cortex has been associated with various neurodegenerative conditions which additionally associate with speech-language symptoms affecting speech rate and/or fluency, including Parkinson’s disease, progressive supranuclear palsy, and others (Barker et al., 2018; Chandregowda et al., 2021; Esmonde et al., 1996; MacDonald & Halliday, 2002; Robinson, 2013; Robinson et al., 2006; Robinson, Spooner, & Harrison, 2015b; Wilson et al., 2010); however, much like in stroke, patterns of neurodegeneration rarely affect one region independently (Harper et al., 2017), and thus distinctions between the SMA proper and the pre-SMA in neurodegeneration are difficult to investigate (cf. MacDonald & Halliday, 2002).

A population that provides a complementary perspective not limited by these same constraints is individuals undergoing resective neurosurgery. Though long-term language outcomes in resective neurosurgery are generally excellent, transient aphasias occur in approximately 70% of left-hemisphere resections in the first week following surgery, providing unique insights into lesion-symptom relationships via uncommonly precise, circumscribed lesions (Wilson et al., 2015). A well-known outcome of neurosurgeries impacting the SMA proper is “SMA syndrome,” characterized by transient global akinesia (i.e., reduction in voluntary motor function) with normal muscle tone and (in cases of resection in the language-dominant hemisphere) akinetic mutism that generally resolves within several weeks of the resection (Mathew et al., 2018; Nakajima et al., 2019; Potgieser et al., 2014). Literature on human neurosurgical injury to the *pre*-SMA specifically, however, is sparse (cf. Satoer et al., 2014).

While the pre-SMA is often included in models of the neural bases of speech (i.e., the motor planning and execution aspects of language production; Bohland et al., 2010; Dick et al., 2019; Price, 2010), it is absent from many of the dominant models of the broader language network (e.g., Dell et al., 2013; Fedorenko et al., 2024; Friederici & Singer, 2015; Hagoort, 2013; Hickok & Poeppel, 2007; Ueno et al., 2011; see Binder et al., 2009; Hertrich et al., 2016, for further discussion of this point). One important exception is an influential model of the semantic system derived from a meta-analysis of functional imaging studies, which draws particular attention to this region as “largely overlooked … despite its consistent activation” and suggests a role relevant to semantic processing (Binder et al., 2009, p. 2777). Detailed neuropsychological testing in patient cohorts has alternatively suggested that this area may instead be crucially involved in energization, a domain-general “process of initiation and sustaining of a response over time” (Barker et al., 2018, p. 350; Robinson et al., 2012; Stuss & Alexander, 2007) that is not specific to linguistic or semantic contexts; however, the extent to which this process may differ between cases of left and right pre-SMA involvement remains somewhat unclear (Barker et al., 2018; Stuss & Alexander, 2007).

Recently, in a large and still-growing neurosurgical cohort at the University of California, San Francisco (UCSF), we have observed several cases of a unique language presentation following left pre-SMA resection—namely, impaired speech production during spontaneously self-generated, but not externally cued, contexts. This language profile is rare, with related syndromes estimated at a prevalence of about 1%–13% across etiologies (Bohra et al., 2015; Crosson et al., 2018; Pedersen et al., 2003). Importantly, this deficit is non-motor in nature; it is not accompanied by hemiparesis or akinetic mutism as in SMA syndrome that results from damage to the SMA proper (Mathew et al., 2018; Nakajima et al., 2019; Potgieser et al., 2014), nor articulatory groping and distortions as observed in apraxia of speech (AOS), that results from damage to more lateral frontal regions (Basilakos et al., 2015; Itabashi et al., 2016; Levy et al., 2023; Silva et al., 2022). Many terms, including *dynamic aphasia* (Akhutina, 2016; Barker et al., 2018; Luria & Hutton, 1977; Robinson et al., 1998), *transcortical motor aphasia* (Goodglass et al., 2001; Kertesz, 2007), *verbal adynamia* (Barker et al., 2021; Magdalinou et al., 2018), and *dysexecutive aphasia*/*syndrome* (Ardila, 2010; Stuss & Alexander, 2007), among others, refer to similar language profiles to the one of interest in this study (see Discussion); to limit theoretical assumptions, we will refrain from strong classification according to existing labels and simply describe the symptoms we observed.

Here, we conduct a retrospective study on a large (*n* = 307) database of neuroimaging and language data following neurosurgical resections at UCSF to probe the neural bases of transient self-generated speech deficits not attributable to motor speech impairment.

To illustrate the nature of this deficit, we highlight the case of Edwin Ko, a professional linguist, coauthor of the present study, and member of this neurosurgical cohort. This case is highlighted here as a representative sample of the syndrome of interest, and due to the unique insights provided by its accompanying first person account (see Supporting Information 2 of the Supplementary Data); transcripts of evaluations from other key cases are included in the Supporting Information 3 of the Supplementary Data.

### Representative Case Description

At the time of his presentation at UCSF, Edwin Ko was a 31-year-old right-handed male presenting with a new onset seizure. A 4.8 × 2.9 × 3.9 cm lesion in the left medial superior frontal gyrus was identified on magnetic resonance imaging. The Quick Aphasia Battery (QAB; Wilson et al., 2018) was administered by a trained language researcher pre-operatively, on which Edwin performed within normal limits.

**Figure 1.**
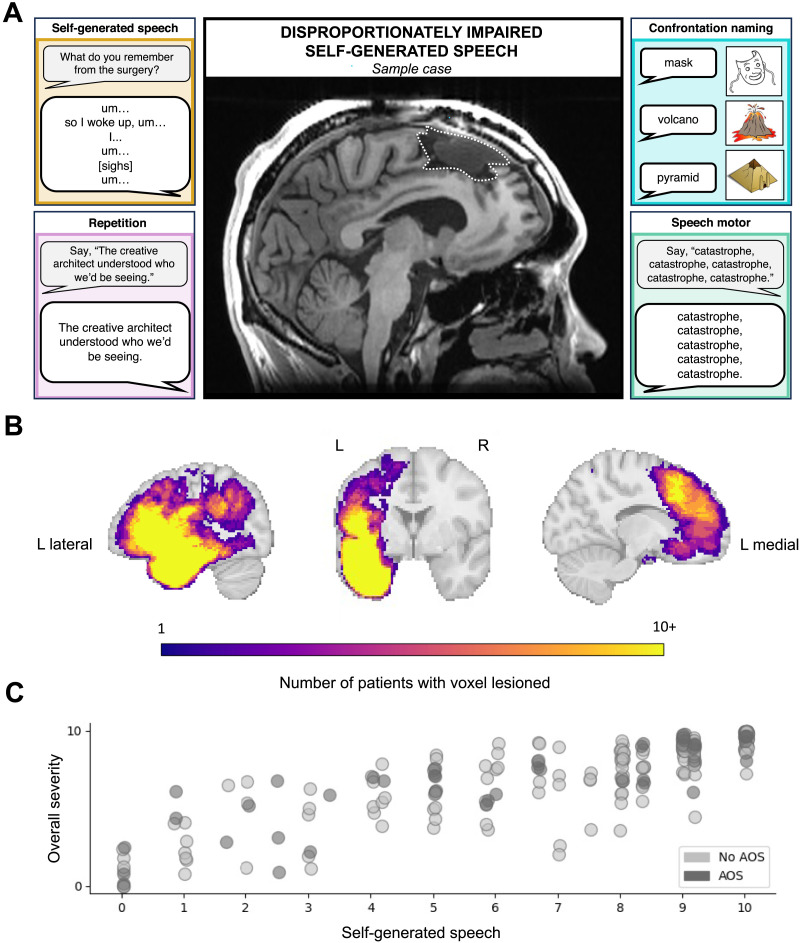
Representative case and characterization of database. (A) Representative case in which self-generated, but not externally cued, speech was impaired. Middle image shows post-operative imaging with resection cavity outlined while outer boxes demonstrate performance on various language tasks. (B) Overlay of resections included in full dataset. (C) Relationship between self-generated speech metric and overall severity in the full *n* = 307 dataset, with 0 corresponding to completely impaired and 10 corresponding to unimpaired. Darker circles = apraxia of speech (AOS) present; lighter circles = no AOS present.

At 2 days post-surgery, Edwin was again evaluated using the QAB (Figure 1A). Connected speech exhibited frequent word-finding pauses, slow rate, and unfinished utterances; a sample utterance is as follows (prompts from the examiner and/or event descriptions are displayed in brackets):

*[can you tell me what you remember from the surgery?] yeah, um …*

*so I … woke up, um …*

*I …*

*um …*

*I don’t know which order this is, but … um …*

*I …*

*had a …*

*picture association (.) [clears throat] task?*

*um …*

*and then I …*

*um …*

*had a …*

*um …*

*…*

*[sighs]*

*um …*

*um …*

*[are you remembering something and it’s hard to get the words for it?]*

*yeah. [laughs]*

When presented with simple pictures to describe, Edwin easily produced syntactically well-formed sentences, such as “The boy is washing the (.) girl” and “The girl is kicking the boy.” He was able to correctly name all items in confrontation naming with minimal hesitation (9.59/10) and correctly repeat all repetition items, including full sentences, aside from one subtle phonemic error (with the word “architect” pronounced as /aɹkitɛkt/ rather than /aɹkətɛkt/: 9.17/10). Comprehension was additionally a strength relative to connected speech production (word comprehension: 10/10; sentence comprehension: 8.75/10). No apraxia of speech (AOS) or dysarthria was observed.

At 1 month post-surgery, Edwin had returned largely to his baseline. A first person account of his experience of recovery is included in Supporting Information 2 of the Supplementary Materials. Some key selections are as follows:

First person:

- “I vividly remember nurses asking me standard questions … [I]f I was asked to rate myself on how I responded, I would give myself a solid A++. It was [only] when … my neuro-oncologist … came to visit that I realized I was experiencing symptoms of aphasia … she asked me to describe what a linguist does. As I tried to respond to her question … I found myself struggling to express my thoughts in words. I thought to myself, *Hmm. Well, that’s odd. Why do I have difficulty describing what I find so much joy doing?* … My comprehension remained fully intact.”
- “[I]t was as if I was a farmer and all the words were buried beneath the soil. I was constantly trying to find that one specific word in the field that contained all these different words. Except I didn’t know where that word was. And so I kept on digging and digging, just trying to locate that one word to no avail.”

Observations from others with professional training in linguistics:

PhD advisor:

- “[I]t very often seemed that [he] had an idea to express, or a thing to say, but couldn’t find it. It was like inside [his] head was a large, large room and [he was] looking for the thing [he] needed and could not see it, though [he] knew exactly what it was … [He was] excellent with yes–no questions, but open-ended questions were not guaranteed to get an answer. … Some vocabulary, which I would have considered very specialized, was accessible just fine. … It felt as if certain pathways or routines were working just fine, even as many or most were closed off.”

Colleague/friend:

- “I remember [him] struggling with lexical retrieval but not with the word [‘lexical retrieval’] … [O]nce a word was activated (that is, once [he] found it or we reminded [him] of it) [he was] able to keep using it during the conversation. … [He] never left out function words … but needed a word bank provided by us in conversation.”

To summarize, Edwin was able to retrieve words when presented with simple images; produce, comprehend, and repeat grammatical sentences; and easily complete motor speech tasks following resection of the pre-SMA. However, he was markedly unable to retrieve the words necessary to express himself in the context of self-generated speech. This behavior is emblematic of the deficit in self-generated speech we aim to investigate here.

## MATERIALS AND METHODS

### Patient Population

Three hundred and seven patients undergoing left-hemisphere surgical resection for tumor, seizure focus, or vascular malformation were included in this study, with inclusion criteria of (1) left hemisphere resection; (2) acute postoperative structural imaging sufficient for 3D reconstruction; and (3) postoperative language evaluation acquired 1–5 days post-surgery. Demographic and clinical information on study participants is detailed in Table 1. Patients gave their written informed consent to participate in this study. The research protocol was approved by the UCSF Committee on Human Research.

**Table 1.** Demographic information

Sex (M/F)	178/129
Age in years (range)	46.21 ± 16.21 (18–87)
Handedness (R/L/A/U)	281/19/4/3
Etiology	
Low-grade glioma	103
High-grade glioma	145
Epilepsy	34
Vascular malformation	14
Brain metastases	9
Necrosis	2
Image used for resection drawing	
T2 FLAIR	249
T1	57
CT	1
Resection size in cm^3^ (range)	21.43 ± 18.60 (0.36–134.03)

*Note*. *Abbreviations*: M = male; F = female; R = right; L = left; A = ambidextrous; U = unknown; FLAIR = fluid-attenuated inversion recovery.

### Resection Delineation

Trained researchers at UCSF delineated resections on post-operative structural imaging of sufficient resolution for 3D reconstruction using MRIcron (Rorden & Brett, 2000) (see Table 1 for imaging details). The resulting 3D masks were smoothed using a 4-mm full width at half maximum (FWHM) Gaussian kernel. Both the original images and masks were then warped to normalized (Montreal Neurological Institute/MNI) space using the unified segmentation procedure in SPM (Ashburner & Friston, 2005) with cost-function masking of the lesion and resampled to a voxel size of 2 × 2 × 2-mm. The most commonly resected regions in this dataset were the left temporal pole, the insula, and the rolandic operculum; medial prefrontal resections were also well represented (Figure 1B).

### Language Evaluation

Language was evaluated by a trained researcher or speech-language pathologist using either the Western Aphasia Battery (WAB; for data collected between 2011 and 2016; Kertesz, 2007) or the QAB (for data collected between 2016 and present; Wilson et al., 2018). The WAB is a validated and commonly used aphasia assessment resulting in an overall severity score, summary scores on various subdomains of language, and an aphasia subtype classification. The QAB is a newer, non-classificatory language assessment which demonstrates high concurrent validity with various subscores of the WAB (Wilson et al., 2018), providing an overall severity score in addition to a language profile across seven language dimensions on a 0–10 scale (0 being the most impaired and 10 being unimpaired). Evaluations were recorded using either audio-only (2011–2021) or audio–visual (2021–present) recordings.

### Conversion Between Language Evaluations

As the QAB and the WAB are non-identical in their assessment properties and scoring mechanisms, a conversion method for putting WAB scores and QAB scores into a single shared space was developed and tested using a separate dataset in which both the QAB and the WAB were conducted in the same set of 14 individuals following stroke (see supplementary materials of Wilson et al., 2018). Given the profile of interest, the primary behaviors we wished to capture were perceived fluency in self-generated contexts, overall linguistic abilities, and presence or absence of speech motor programming deficits (i.e., AOS). To quantify fluency in self-generated contexts, we used the Fluency rating from the spontaneous speech tasks on the WAB (0–10 scale) and the average of the Reduced Length and Complexity, Reduced Speech Rate, and Overall Communication Impairment ratings from the QAB’s connected speech tasks (rescaled from a 0–4 scale to a 0–10 scale); correlation between the two metrics in the independent dataset was strong at *r*(13) = 0.91, *p* < 0.001. (Note that the WAB’s measure of fluency has been shown to capture various dissociable dimensions of speech production, including logopenia [i.e., paucity of speech], agrammatism, and motor speech deficits (Casilio et al., 2019); the QAB metrics selected here map best onto those logopenic dimensions, such as pauses, abandoned utterances, and reduced speech rate, that characterize the non-motor, non-syntactic deficit of interest in this study.) To quantify overall language abilities, we used the Aphasia Quotient from the WAB (rescaled from a 0–100 scale to a 0–10 scale) and the QAB Overall score (0–10 scale); correlation between the two metrics in the independent dataset was again strong at *r*(13) = 0.94, *p* < 0.001. Presence or absence of speech motor programming deficits (which are not rated in the standard form of the WAB) was assessed based on perceptual impression in a consensus procedure between four expert raters (two PhDs in language neuroscience, one neurosurgery resident, and one speech-language pathologist) for a separate study in 2020. For a representation of how these three scores relate to each other in our dataset, see Figure 1C. To contextualize these scores, we additionally developed shared-space metrics for repetition (WAB: Repetition score, QAB: Repetition summary score; *r*(13) = 0.95, *p* < 0.001) and confrontation naming (WAB: Object Naming rescaled to 0–10, QAB: Picture Naming rescaled to 0–10 [note that scores from the QAB’s Picture Naming task, rather than the overall Word Finding summary score, was used due to its greater correspondence to the Object Naming task on the WAB, which does not reflect anomia in connected speech]; *r*(13) = 0.94, *p* < 0.001); these measures are included primarily for descriptive purposes and not used directly in the analyses described below.

### Analysis

VLSM 2.55 (https://aphasialab.org/vlsm/; Bates et al., 2003) was used to identify systematic relationships between locations of resection and fluency in self-generated speech at the acute time point. Voxel-based lesion-symptom mapping (VLSM) is a mass-univariate method that fits a general linear model at each voxel to quantify the relationship between lesion status and behavioral outcome. As our primary behavioral outcome of interest was the striking ability to fluently produce language in task-constrained, but not self-generated, contexts, we treated our metric of fluency in self-generated contexts (i.e., self-generated speech) as our behavioral outcome of interest. While this measure is not perfect (see, e.g., Casilio et al., 2019, for a breakdown of various possible subdomains within the notion of “fluency”), it was selected for its utility of being easily quantifiable and aligning well with our perceptual impressions of the profile (see Supporting Information 3 of the Supplementary Materials for sample evaluation transcripts and corresponding scores on the self-generated speech metric). Covariates included overall language abilities, presence of AOS, language evaluation (WAB or QAB) used, days post-surgery at which evaluation was collected, and lesion size, to avoid spurious results attributable to global language deficits, motor speech deficits, assessment differences, recovery time, and overrepresentation of specific voxels in large lesions, respectively. The threshold for voxel inclusion was set to 5 (i.e., each voxel had to be lesioned in at least 5 patients to enter the analysis) and 1,000 iterations were used for permutation testing (Wilson et al., 2015). As the pathological processes at play differ in gliomas, vascular malformations, metastases, and epilepsy, a second VLSM was also conducted in which only glioma patients were included (*n* = 244).

A complementary analysis—support vector regression-based multivariate lesion symptom mapping (SVR-MLSM; https://github.com/atdemarco/svrlsmgui; DeMarco & Turkeltaub, 2018)—was completed to account for some known limitations of univariate VLSM (Mah et al., 2014; Nachev, 2015). In contrast with the univariate VLSM method, MLSM considers many voxels at once in a single multivariable regression model that projects the model weights back onto the brain, rather than conducting separate statistical tests within each voxel. In this analysis, the same outcome score (fluency in self-generated speech), covariates (overall language abilities, lesion size, presence of AOS, evaluation used, and days post-surgery of evaluation), threshold for voxel inclusion (*n* = 5), and number of permutations (1,000) as the VLSM analysis were used, with nuisance variables covaried out of the behavioral outcome and direct total lesion volume control used for lesion volume correction.

## RESULTS

### VLSM

**Figure 2.**
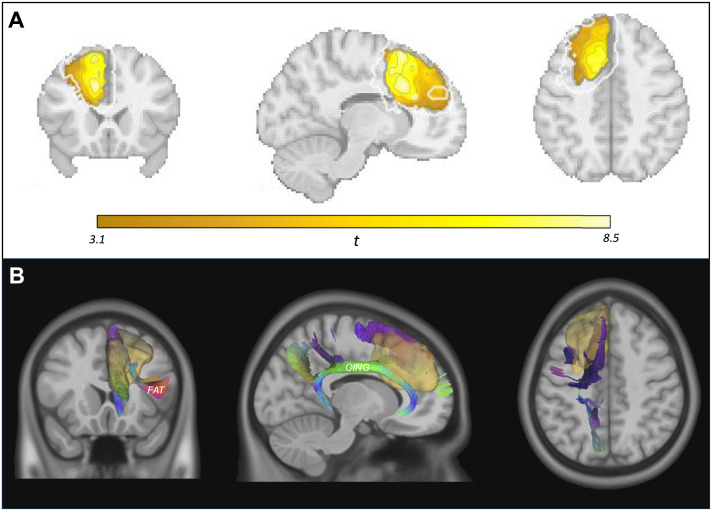
Neural correlates of disproportionate deficit to self-generated speech. (A) Region identified using lesion-symptom mapping (LSM) analyses; resection of colored voxels was significantly correlated with decreased fluency despite relatively preserved overall language and speech-motor abilities. Dark gray outline corresponds to border of voxel-based LSM cluster; white outline corresponds to border of multivariate LSM cluster. (B) White matter tracts intersecting with the LSM-based region of interest (yellow/opaque). FAT = frontal aslant tract; CING = cingulum.

A single significant cluster of voxels (53.36 cm^3^) systematically related to deficits in self-generated speech was localized to the left medial prefrontal cortex, with the center of mass of the volume falling in the medial superior frontal gyrus (MNI coordinates −18, 23, 39; Figure 2A). The VLSM results were essentially identical in the *n* = 244 glioma-only analysis, identifying a slightly smaller region (40.14 cm^3^) with its center of mass located 8.72 mm away from that of the original cluster; Dice similarity between the two clusters was 0.86.

### SVR-MLSM

Broadly consistent with the results found using VLSM, a single, albeit larger, significant cluster of voxels (117.04 cm^3^) was again localized to the left medial prefrontal cortex, with the center voxel in this case falling in the frontal aslant tract (MNI coordinates −21.2, 15.8, 39; Figure 2A). The Dice similarity coefficient (DSC) between the VLSM and the MSLM cluster was 0.52, suggesting moderate overlap; see Ivanova et al. (2021), for further elaboration on general similarity between VLSM and SVR-MLSM results when spatial localization of symptoms is the investigative aim.

As the VLSM analysis and the SVR-MLSM analysis gave us similar but non-identical findings, we calculated the intersection of the clusters from each analysis and defined this as our lesion-symptom-mapping-based region of interest (LSM-ROI) for use in follow-up analyses. Per intersection with a custom combined gray and white matter atlas (based on Rolls et al., 2020; Warrington et al., 2020), this region fell primarily in the following regions: the frontal aslant tract, medial superior frontal gyrus, pre-SMA, superior frontal gyrus, and perigenual cingulum (Figure 2B; Yeh et al., 2010).

### Follow-Up Analyses: Relative Risk

**Figure 3.**
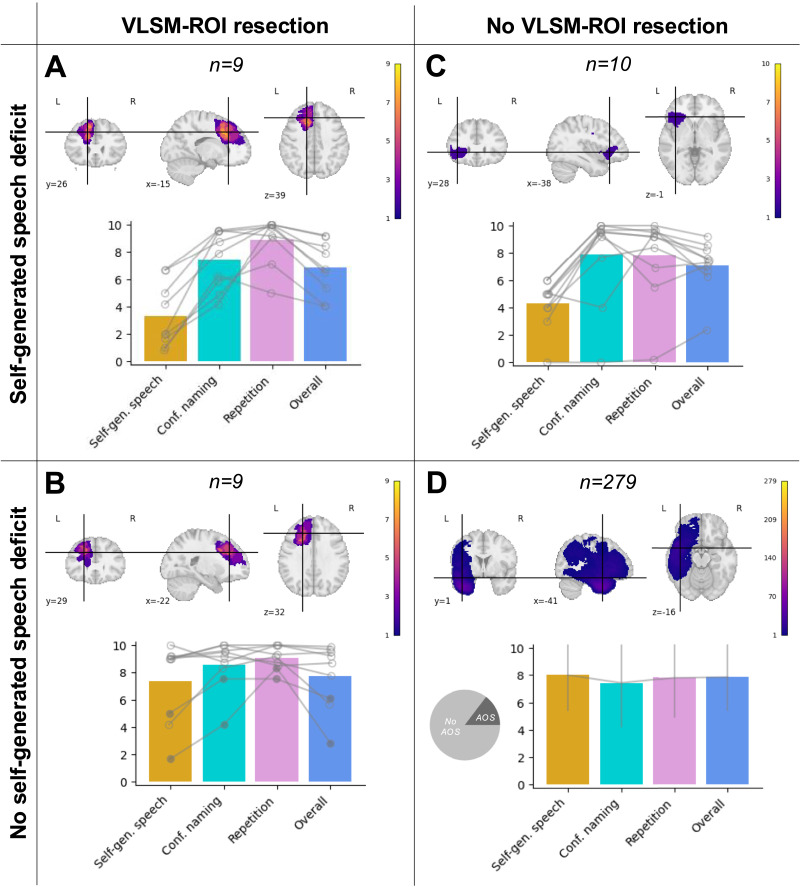
Contingency table of self-generated speech deficit versus LSM-ROI. Columns = patients with a resection similar to (A, B) versus not similar to (C, D) the area related to the LSM-ROI. Rows = patients with a speech deficit corresponding to (A, C) versus not corresponding to (B, D) the self-generated speech deficit. Filled circles = apraxia of speech present. Error bars correspond to standard deviation. AOS = apraxia of speech.

In order to demonstrate that the region identified in our LSM analyses (LSM-ROI) did indeed correspond to real-world resections (as opposed to simply reflecting the voxelwise overlap of many disparate lesions), a relative risk analysis of the relationship between those best described as having LSM-ROI resections and those best described as having disproportionate deficits to spontaneous speech was conducted. Participants with LSM-ROI resections were defined as those whose resections had a DSC with the LSM-ROI of greater than or equal to 1.5 standard deviations from the mean in the dataset (*n* = 18; Figure 3A, 3B). Participants with disproportionate deficits to spontaneous speech were defined as those patients in whom the difference between their overall severity score and their spontaneous speech score was greater than or equal to 1.5 standard deviations from the mean in the dataset, with the additional stipulation that no apraxia of speech be present (*n* = 19; Figure 3A, 3C). There was a significant difference in the relative risk of having a disproportionate self-generated speech deficit following an LSM-ROI resection compared to a resection elsewhere (RR = 14.45, 95% CI [6.73, 31.03]); that is, participants were nearly 15 times more likely to present with the self-generated speech deficit following a resection in the area identified using LSM than a resection at a different location in the brain. There was no clear locus of lesion overlap in the patients without LSM-ROI resections who presented with the self-generated speech deficit (Figure 3C; note that region of maximum overlap consists of only three individuals). A complementary odds ratio analysis similarly demonstrated a positive association between the self-generated speech deficit and LSM-ROI resections (OR = 27.90, 95% CI [9.11, 85.40]). For a sense of the behavioral presentation and to see how the self-generated speech metric maps onto behavior in the *n* = 9 patients with the deficit following LSM-ROI resection (Figure 3A), see Supporting Information 3 of the Supplementary Materials.

As not all patients with LSM-ROI resections presented with the self-generated speech deficit, we aimed to better understand this variability. Thus, we probed for any differences between those with and without the disproportionate self-generated speech deficit within the *n* = 18 individuals with LSM-ROI resections (Figure 3A, 3B) across nine variables: etiology, evaluation used, days post-surgery of age, total resection size, and percent damage to each of the regions most represented in the LSM-ROI (namely, the frontal aslant tract, medial superior frontal gyrus, pre-SMA, superior frontal gyrus, and perigenual cingulum). A series of Pearson’s chi-square tests of independence suggested no significant association between etiology (high-grade glioma, low-grade glioma, vascular malformation, or epilepsy) and presence of the self-generated speech deficit (*χ*^2^(3, *N* = 18) = 2.25, *p* = 0.32), nor any significant association between evaluation used (WAB, QAB) and presence of the self-generated speech deficit (*χ*^2^(1, *N* = 18) = 0.00, *p* = 1.00). Rank-sum tests suggested greater damage to the pre-SMA in those with the self-generated speech deficit compared to those without it (U = 109, *p* = 0.03) and a marginal but non-significant effect of the frontal aslant tract (U = 107, *p* = 0.06); none of the other variables showed significant differences across the groups (days post surgery: U = 88, *p* = 1.00; lesion size: U = 79, *p* = 0.60; age: U = 76, *p* = 0.42; medial superior frontal gyrus: U = 96, *p* = 0.37; superior frontal gyrus: U = 84, *p* = 0.93; perigenual cingulum: U = 94, *p* = 0.44).

Finally, of the *n* = 18 patients with LSM-ROI resections, *n* = 10 had follow-up language evaluations available at 1 month or later post-surgery. While all participants showed marked improvement in this time frame, 2/13 still did not reach language within normal limits per QAB criteria (Wilson et al., 2018) at 1 month post-surgery; in all cases this deficit was driven by disproportionate impairment to spontaneous speech (see Supporting Information 1 of the Supplementary Data, sample case 5).

## DISCUSSION

Making use of a large (*n* = 307) database of individuals with left hemisphere surgical resections, we found evidence to support a systematic relationship between damage to left dorsomedial prefrontal regions and non-motor-speech deficits in spontaneously self-generated, but not externally cued, language production.

We first established this relationship using both a mass-univariate and a multivariate LSM analysis, in which the degree of self-generated speech deficit was modeled as variation in fluency holding overall impairment and speech motor abilities constant. These analyses revealed a single significant cluster of voxels in the dorsomedial prefrontal cortex that was strongly related to the degree of self-generated speech deficit observed (that is, more damage to the LSM-ROI led to lower fluency scores, when accounting for overall impairment and motor speech abilities). We then observed that the self-generated speech deficit emerged nearly 15 times more frequently in the context of resections in the LSM-ROI than it did in the context of other resections. Among considered variables (i.e., etiology, age, total resection size, or percent damage to each of the regions the LSM-ROI overlapped), only damage to the pre-SMA showed a significant relationship with the presence of the self-generated speech deficit. For this reason, we call specific attention to the pre-SMA in the remainder of the discussion.

### Prior Literature on the Pre-SMA

The role of the pre-SMA in humans remains elusive, in part due to a lack of clarity on its anatomical and functional boundaries (Clairis & Lopez-Persem, 2023; Dadario et al., 2023; Ruan et al., 2018). While the SMA proper is known to project directly to the primary motor cortex and spinal cord and is believed to contain somatotopic representations of the body (Eickhoff et al., 2011; Potgieser et al., 2014), the relationship of the pre-SMA to motor execution is less clearcut. Rather, the pre-SMA and its neighboring regions have been associated with more abstract functions relevant for action, that is, supporting complex condition–action associations (Aquino et al., 2023), determining how and/or whether to act or stop acting (Swann et al., 2013; Zapparoli et al., 2018), and encoding the will to persevere when action is perceived to be challenging (Fried, 2022; Parvizi et al., 2013). In functional neuroimaging paradigms, the pre-SMA (and nearby regions often reported under this same name; see Clairis & Lopez-Persem, 2023; Dadario et al., 2023) often shows activations that scale with attentional and/or task demands, as described in characterizations of the salience (Seeley, 2019) and multiple demand (Duncan, 2010; Fedorenko et al., 2013; Quillen et al., 2021) networks; pre-SMA activity has additionally been associated with task paradigms examining semantic processing (Binder et al., 2009; Lambon Ralph et al., 2017). Additionally, one PET study demonstrated increased involvement of the left pre-SMA region in propositional (i.e., responding to autobiographical prompts) versus non-propositional (i.e., counting) speech tasks (Blank et al., 2002), though recitation of overlearned nursery rhymes also activated the pre-SMA in this case. In non-human primates, studies of pre-SMA suggest it is modulated by memory-based motor sequence execution (Shima & Tanji, 1998), action selection/task-switching (Hikosaka et al., 2019; Isoda & Hikosaka, 2007), and motor sequence learning (Hikosaka et al., 1995; Kalivas & Nakamura, 1999; Shima & Tanji, 1998), proposed to be mediated by circuits from pre-SMA to the basal ganglia through the subthalamic nucleus (Hikosaka et al., 2019; Isoda & Hikosaka, 2007).

In line with these findings, one theory suggests that the pre-SMA acts in tandem with the reward system in the striatum to handle response conflict, assigning weights to particular actions for selection (Nachev et al., 2007), while another suggests that it plays a role in energization, or the ability to sustain an ongoing response in the absence of external cues (Robinson et al., 2012; Stuss & Alexander, 2007). Either of these interpretations may be consistent with findings described here, in that there is a greater need to both (a) internally mediate between verbal actions when the space of possibilities is greater, and/or (b) maintain an ongoing responsive thread without external cueing in spontaneous, self-generated speech compared to constrained language tasks. We were unable to probe either of these hypotheses in detail due to the retrospective nature of the study; however, prior neuropsychological work has supported both hypotheses in a clear behavioral analog to what we observed here (Robinson et al., 1998, 2005, 2010, 2012, 2015b, 2021), described in detail below.

### Related Behavioral Syndromes

Deficits in producing self-generated, but not externally cued, language have been reported and theorized under many names throughout the history of aphasiology.

Transcortical motor aphasia (TMA), defined primarily by preserved repetition in the absence of fluent speech, is a well-established syndrome similar to what we observed here, and has often been associated with damage to medial prefrontal regions (Crosson et al., 2018; Freedman et al., 1984). However, TMA is most often diagnosed using the WAB or the Boston Diagnostic Aphasia Examination (BDAE; Goodglass et al., 2001), neither of which account for any potential motor-speech deficits affecting spontaneous production in their classification schemes. Previous work on the lesion bases of TMA may therefore be confounded by the presence of AOS affecting speech output, as well as other limitations associated with criterion-based aphasia classifications (Casilio et al., 2019; Wertz, 1983).

In personal correspondence, Edwin stated, “Anomia [is the diagnosis] I mostly was drawn to, because I was experiencing some level of difficulty retrieving words, but I couldn’t find a particular kind of aphasia that really [fit what I was experiencing]”. In line with this observation, the term “anomia” is used clinically in at least two different ways: one, to describe a *symptom*—that is, difficulty retrieving desired words, regardless of overall aphasia severity (Bonilha et al., 2015; Casilio et al., 2019; Nardo et al., 2017)—and the other, to describe a *syndrome*—that is, a diagnosis on a classificatory language assessment in which language is mildly impaired overall and word retrieval is the primary deficit (Goodglass et al., 2001; Kertesz, 2007). While individuals with the self-generated speech deficit observed here would be too nonfluent to meet criteria for anomia as a *syndrome*, many characteristics of the *symptom* of anomia (e.g., abandoned utterances, word-finding pauses, comments on inability to find words; see Casilio et al., 2019) were present in their connected speech. This is in stark contrast to their relatively preserved ability to retrieve words in a confrontation naming task (see Representative Case Description, Figure 1A). However, lexical retrieval was not probed in detail in the majority of these patients beyond the relatively simple probes in the WAB or the QAB, and many other characteristics of anomia, such as circumlocutions and other strategies for self-cueing, were not observable in these patients’ very minimal connected speech (see Supporting Information 1 of the Supplementary Data). Difficulties in naming can occur due to disruptions at various levels of processing (e.g., phonemic, lexical, semantic), and have therefore historically been very difficult to localize (Levelt et al., 1999; Sheppard & Sebastian, 2021). Thus, we do not have sufficient justification to characterize the self-generated speech deficit observed here as anomic in nature.

The profile described here is perhaps most consistent with Luria’s dynamic aphasia, described as “a distinctive ‘lack of spontaneity of speech’ … [wherein] the fundamental disturbance … appears when [patients] change over from repeated or habitual speech to independent expression” (Luria, 1964, p. 159). Dynamic aphasia has been described in both stroke (Bormann et al., 2008; Cox & Heilman, 2011; Crescentini et al., 2008; Gold et al., 1997) and non-stroke populations, including individuals with tumor (Costello & Warrington, 1989; Robinson et al., 1998; Satoer et al., 2014), neurodegenerative disease (Barker et al., 2018; Chandregowda et al., 2021; Esmonde et al., 1996; Robinson, 2013; Warren et al., 2003), and genetic malformations (Barker et al., 2021; Berthier et al., 2020). Dynamic aphasia has been associated with damage to medial frontal regions as well as inferior frontal regions, with some suggesting nuanced differences in behavioral presentation across the regions damaged (Barker et al., 2018; Crosson et al., 2018; Robinson et al., 2012). Often this profile is conceptualized as occurring at the boundary of linguistic and cognitive skills, as a deficit in verbal executive functioning or the inner organization of speech (Akhutina, 2016; Barker et al., 2018, 2020). The literature on the neural bases of dynamic aphasia has largely been dominated by single case studies or series. Two exceptions, Robinson et al. (2012) and Robinson et al. (2010), in which a series of sentence generation (contrasting high- and low-competition cues) and fluency (contrasting various verbal and non-verbal fluency) tasks were conducted in a dataset of *n* = 67 patients with varying lesion locations, included 18 left frontal and 12 superior medial patients; a recent study found similar results in an expanded dataset (Robinson et al., 2021). Together, these studies suggested that the left inferior frontal gyrus (IFG) may play a role in selecting between competing conceptual propositions while superior medial regions play a role in energization (that is, “the process of initiation and sustaining of any response,” be it verbal or non-verbal in nature; Stuss & Alexander, 2007, p. 903). Our findings regarding the association of left medial superior frontal regions with deficits in spontaneous speech are largely consistent with these energization findings in that the production of self-generated continuous speech requires both the initiation of a response and the maintenance of attention to that ongoing response in the absence of external cues (Barker et al., 2018). However, our assessments do not directly allow us to disentangle between selection and energization processes, as the spontaneous speech metric reported here may reflect either/both the ability to initiate and sustain attention during a response and/or the ability to select between multiple competing verbal response options. It is notable, however, that the left IFG was not associated with self-generated speech deficits in the present study, despite the dataset having sufficient representation of the IFG to detect deficits in this region (Figure 1B).

The profile described here importantly differs from both SMA syndrome—characterized by transient mutism and hemiparesis following resection in the left SMA proper (Potgieser et al., 2014)—and AOS—characterized by difficulty initiating speech and performing articulatory movements in speech contexts (Strand et al., 2014)—in that it is by definition non-motor in nature. Abulia—that is, lack of motivation—was additionally not present in these cases, as all patients sincerely engaged with the evaluations and attempted to communicate with the examiners.

### Recovery

In most cases, the self-generated speech deficit observed resolves to within normal limits within a month. This finding may suggest high plasticity or redundancy of medial frontal functions, such that its function can be rapidly compensated for in the case of injury (Ardila, 2020). It is important to note, however, that this syndrome does not always resolve so quickly; in at least one of the participants with the self-generated speech deficit observed here, expressive deficits persisted for several months post-surgery. Given the highly specific nature of speech contexts that reveal this deficit (i.e., spontaneous as opposed to constrained tasks), in the context of awake neurosurgery, particular stimulation mapping tasks (e.g., elicitation of spontaneous connected speech) may be necessary to prevent unanticipated deficits.

### Implications for Conceptions of the Language Network

Given the retrospective nature of this study, we cannot unequivocally claim that these symptoms are specific to language, not affecting other cognitive or motor processes. Indeed, other work implicating the pre-SMA in action and decision-making processes in humans and primates suggest that its role is likely more domain general, involved in complex condition-action associations and/or monitoring of response conflict. However, it is undeniable that this region has a clear and striking influence on language production when it is damaged. How, then, should we situate this region in the context of the neural organization of language?

While a number of neurobiological models of language production do include the pre-SMA and other medial prefrontal regions in their conceptions of the language network, the roles ascribed to this region differ greatly among them. Indefrey and Levelt (2004) report the medial frontal gyrus and anterior cingulate to be associated with word fluency, but not picture naming, tasks, consistent with a role in response selection for production; Binder et al. (2009, p. 2777) theorizes the left dmPFC to affect “self-guided, goal-directed retrieval of semantic information”; the GODIVA model suggests a role for the pre-SMA in articulatory planning, specifically in “encod[ing] structural ‘frames’ at an abstract level” (Bohland et al., 2010, p. 1507); Price et al. (2004) implicates the pre-SMA in selection and execution specifically for overt oral motor movements, regardless of whether or not they are for speech; Duffau et al. (2014) associates the pre-SMA and frontal aslant tract (FAT) with executive control; and Dick et al. (2019, p. 148) theorizes that the FAT “plays a domain general role in the planning, timing, and coordination of sequential motor movements through the resolution of competition among potential motor plans,” with left-hemisphere specialization for speech. The majority of these models conceptualize the pre-SMA as an articulatory/motor-speech-associated region, while many of the dominant neurobiological models of language production do not include the pre-SMA or other medial prefrontal regions at all (e.g., Dell et al., 2013; Fedorenko et al., 2024; Friederici & Singer, 2015; Hagoort, 2013; Hickok & Poeppel, 2007; Ueno et al., 2011; see Binder et al., 2009, and Hertrich et al., 2016, for further discussion on this point).

Our observations here fit best with a non-articulatory account of functions in the left pre-SMA, given the absence of motor speech impairment in the vast majority of pre-SMA resections in this study. Indeed, prior work suggests that speech motor planning has its bases in more lateral regions of motor cortex (Basilakos et al., 2015; Itabashi et al., 2016; Levy et al., 2023; Silva et al., 2022). Our findings are better aligned with those of prior work theorizing either that the pre-SMA, through its structural connections with the reward system in the basal ganglia, is involved in weighting the appropriateness of given verbal responses such that no response rises to the level of selection when the pre-SMA is injured (Nachev et al., 2007), or that it is involved in a process of energization, in which an ongoing response must be maintained in the absence of external cues (Robinson et al., 2012; Stuss & Alexander, 2007; Stuss et al., 1998). Due to the relatively coarse-grained behavioral testing completed in this retrospective study, we are unable to disentangle these two theories in detail here. However, it is clear that there is a need for future work investigating the mechanistic properties of this region in both linguistic and non-linguistic tasks, particularly those requiring self-generation of linguistic content in the context of spontaneous speech, to better understand its functional role during healthy speech.

### Limitations

As the population of interest in this study were individuals requiring neurosurgery, language may have been atypically organized prior to surgical intervention, limiting generalizability of our findings. However, reorganization of the language system appears to be relatively infrequent even within clinical populations (Diachek et al., 2022). Furthermore, every patient who presented with non-motor self-generated speech deficits following a pre-SMA resection underwent surgery to treat high-grade glioma (World Health Organization rating ≥ III), suggesting a fast-growing tumor with minimal time for neoplastic reorganization prior to surgery. While postoperative cognitive impairment is a potential confound (Berger et al., 2015), the self-generated speech deficit of interest was uncommon in the post-surgical population, suggesting it is not simply a global effect of surgery; additionally, the lucidity with which the experience of this language deficit is remembered per the first-hand testimony reported herein (see Supporting Information 2 of the Supplementary Data) suggests that, at least in his case, cognition was relatively unimpaired at the time of assessment. Given the predominantly retrospective nature of the study, the relative transience of the deficit of interest, and the use of brief assessment tools (Robinson, Biggs, & Walker, 2015a), we were unable to comprehensively assess the extent to which this deficit is language-specific versus cognitive–linguistic in nature. However, as stated above, the striking impact of resections in this region on language remains clear. We therefore intend to keep accruing data for this dataset, including obtaining electrophysiological measures of this region’s function prior to resection, so that we can more deeply probe these questions in the future.

### Conclusion

In summary, we have demonstrated that damage specific to the dmPFC is associated with a unique kind of aphasia in which internally generated, but not externally cued, language production is impaired, in the absence of motor speech or motivational deficits. This finding is important scientifically, as it serves as one of the largest studies of the neural bases of volitionally generated speech in humans, and does so in a rare and uniquely informative neurosurgical cohort. It is also, however, relevant clinically, as it serves as evidence to practicing neurosurgeons that this region may deserve special attention during surgical procedures, and provides a valuable person-centered narrative of the experience of aphasia that is informative to clinicians across the spectrum of care. In summary, this work establishes the importance of medial prefrontal regions for fluent volitional language production, and suggests that these regions warrant further attention in investigations of the neural bases of language.

## ACKNOWLEDGMENTS

We thank the many neurosurgical patients and their loved ones who generously gave their time to make this work possible; the many participating clinicians and researchers at University of California, San Francisco who aided in the development of this database; Gail Robinson for extremely useful and constructive feedback at the Annual Conference of the Society for the Neurobiology of Language, 2023; three anonymous reviewers for helpful feedback on an earlier version of this paper; and our various collaborators and friends, in particular Yizhen Zhang, Jessie Liu, Jon Gauthier, Nina Dronkers, Maria Ivanova, Alexis Pracar, Nicoletta Biondo, Marianne Casilio, Anna Kasdan, Isaac Pedisich, Matthew Leonard, Ilina Bhaya-Grossman, and Terri Scott for their valuable insights and efforts that aided in this work.

## FUNDING INFORMATION

Deborah Levy, National Institute on Deafness and Other Communication Disorders (https://dx.doi.org/10.13039/100000055), Award ID: F32 DC020096. Edward F. Chang, National Institute of Neurological Disorders and Stroke (https://dx.doi.org/10.13039/100000065), Award ID: U01 NS098971.

## AUTHOR CONTRIBUTIONS

**Deborah Levy**: Conceptualization; Data curation; Formal analysis; Funding acquisition; Investigation; Writing – original draft. **Quinn Greicius**: Conceptualization; Formal analysis; Investigation; Writing – original draft; Writing – review & editing. **Catherine Wang**: Data curation; Investigation; Writing – original draft; Writing – review & editing. **Edwin Ko**: Investigation; Writing – original draft; Writing – review & editing. **Duo Xu**: Writing – original draft; Writing – review & editing. **John Andrews**: Data curation; Supervision; Writing – review & editing. **Edward F. Chang**: Conceptualization; Funding acquisition; Investigation; Supervision; Writing – original draft; Writing – review & editing.

## DATA AND CODE AVAILABILITY STATEMENTS

The data that support the findings of this study are available on request from the corresponding authors. The data are not publicly available because they could compromise research participant privacy and consent. Original code is available on GitHub at https://github.com/deb-levy/dmPFC_NoL.

## Supplementary Material






