# Supplementary material for: Role for Left Dorsomedial Prefrontal Cortex in Self-Generated, but not Externally Cued, Language Production
Source: Neurobiol Lang (Camb). 2025 Jun 12;6:nol_a_00166. doi: 10.1162/nol_a_00166 (PMC12170450; doi:10.1162/nol_a_00166)

Specific part of brain linked to speaking on your own

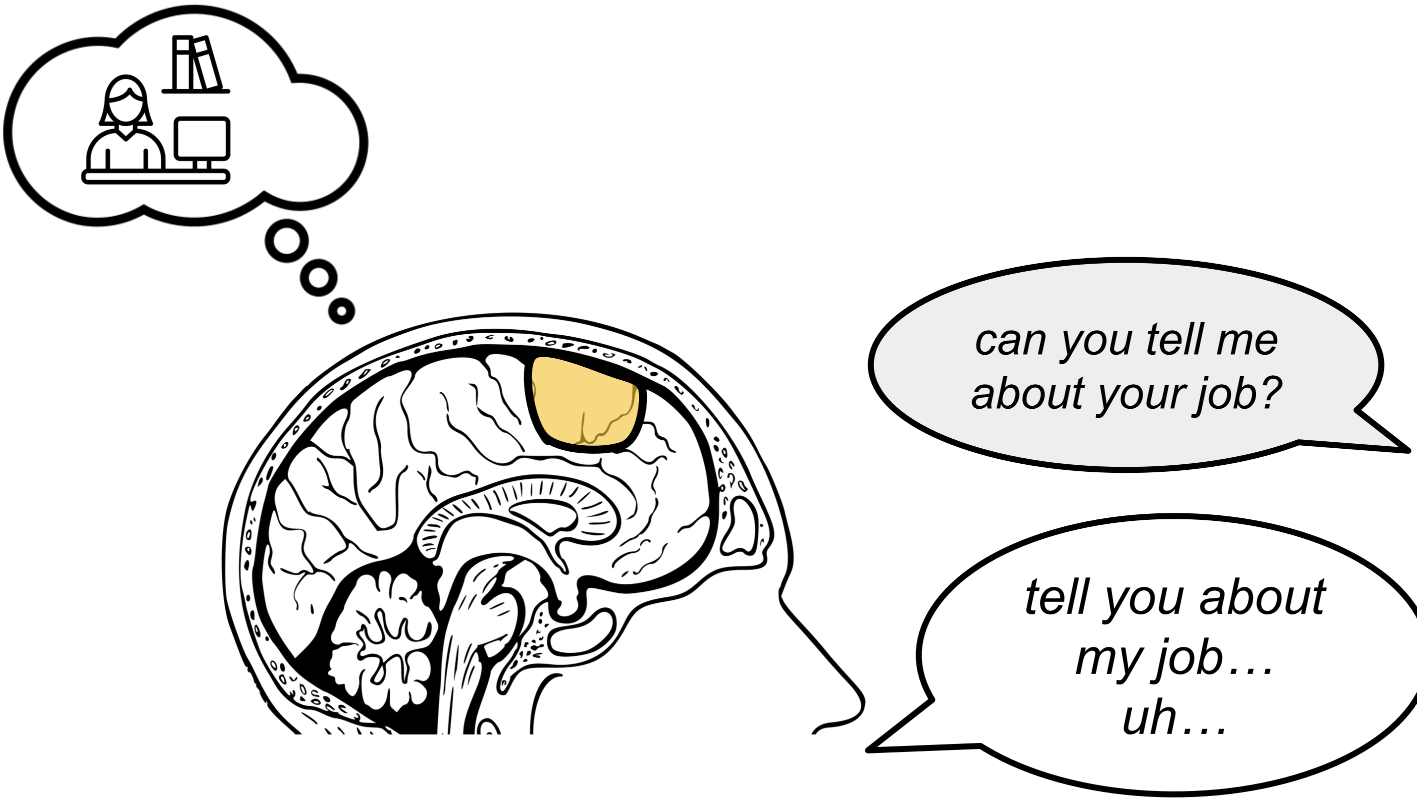

Supplementary Figure 1: Aphasia-friendly manuscript.
An accessible version of "Role for left dorsomedial prefrontal cortex in self-generated, but not externally-cued, language production" designed for individuals with aphasia and their loved ones.

Abstract / Summary

- This study is about a special **part** of the **brain** called the **dmPFC** or **pre-SMA**

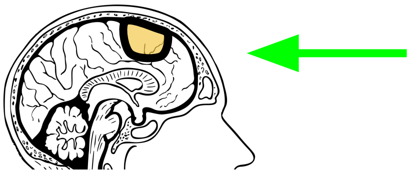

- We looked at a **big group** of **people** who had **brain surgery**

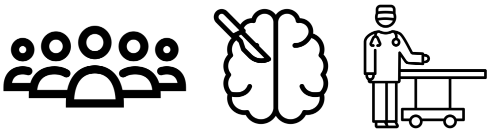

- **Many people** had **trouble talking on their own** after **surgeries** in this **brain area**

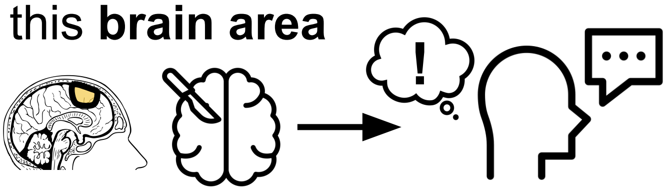

- They had **less trouble talking** when it was **clear** what they were **supposed** to **say**

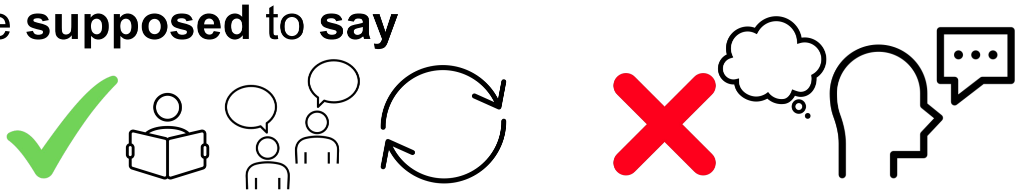

- These people **wanted to talk**, and did **not** have **trouble moving** their **mouths**

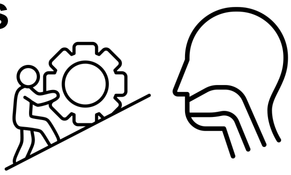

- This taught us that the **dmPFC / pre-SMA** is likely important for **speaking on your own**

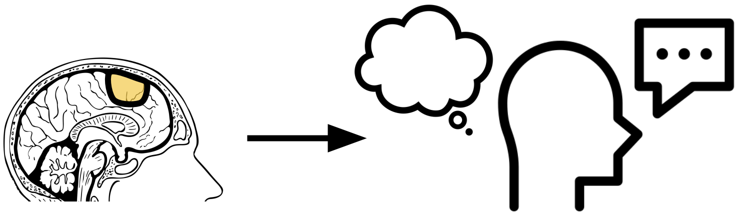

Introduction:
The middle of the brain and language

- This paper is about an area in the **middle** of the **brain** on the **left**

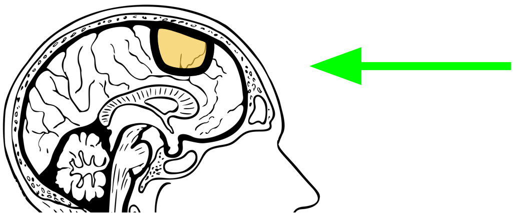

- **Scientists** often **study** this **area**, but there is a lot of **debate** about what it does

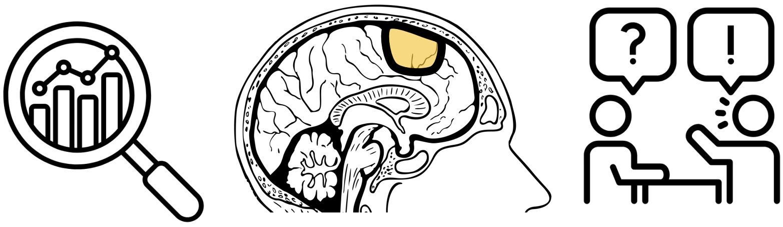

- In the last few years, we saw **a lot** of **patients** who had **trouble talking** after **brain surgeries** in this **area**

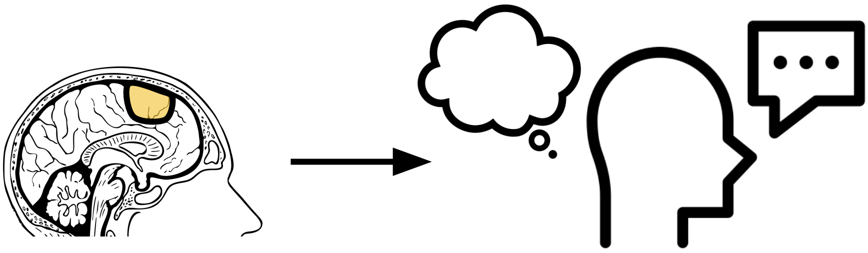

Representative case description:
An example patient

- Here is an example of a person who had surgery in this area:

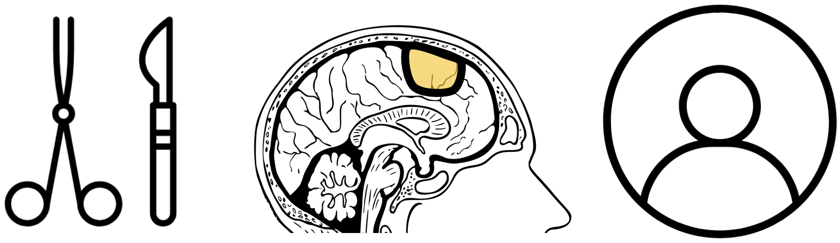

- This person **could**: 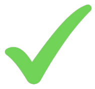
    - **Answer** questions
    - **Describe** pictures
    - **Read** and **repeat** language
    - **Move** his **mouth**

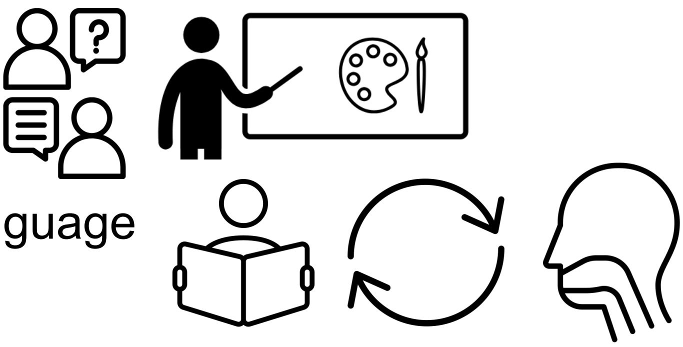

- **HOWEVER**, this person had a lot of **trouble** when he had to **talk on his own**

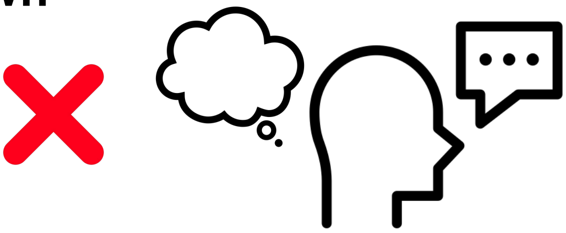

- To **understand** this **better**, we studied a **big group** of people who had **brain surgery**

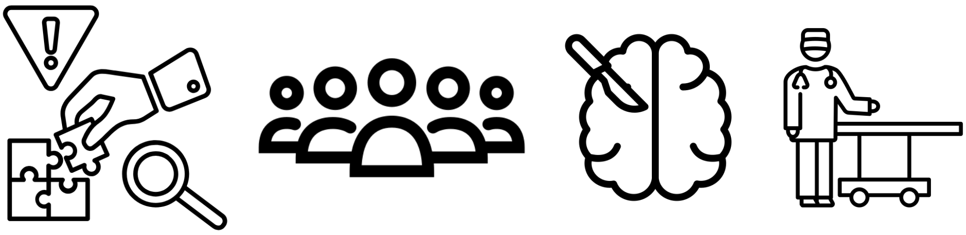

Methods:
Using math to study talking in the brain

- We met **307 people** right after **brain surgery**

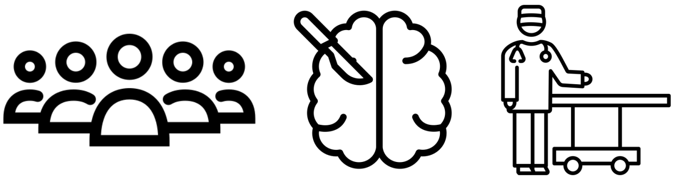

- We got **permission** to include them in this study

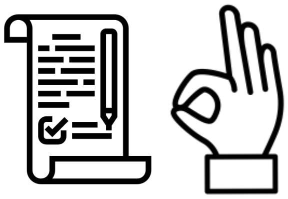

- We used a **computer program** to draw the place of each person’s **surgery**

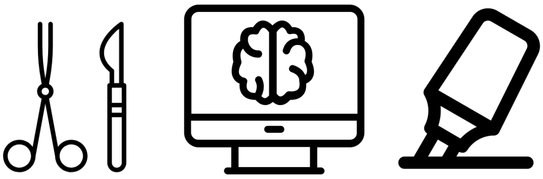

- We **tested** each person’s **language** and made a **score** of how well they could **talk on their own**

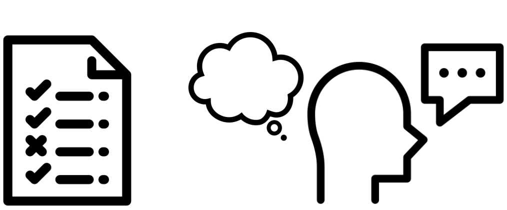

- We used a **special** type of **math** to find the parts of the **brain** that were **most important** for **talking alone**

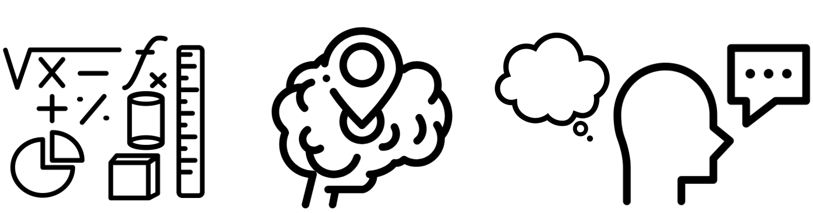

Results:
What we found

- The area in the **middle** of the **brain** on the **left** was the **most important** for **talking alone**

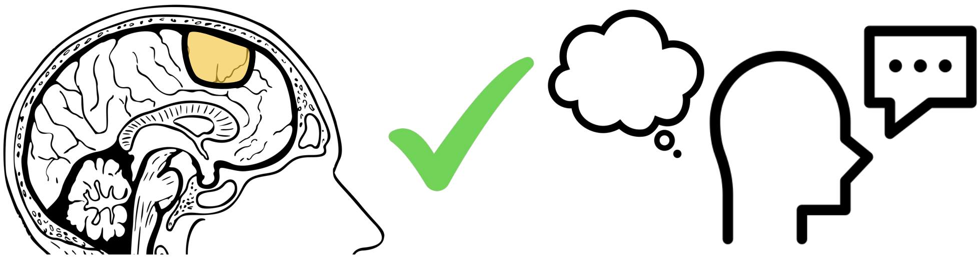

- About **half** of people with **surgeries** in this **area** had specific trouble **talking independently**

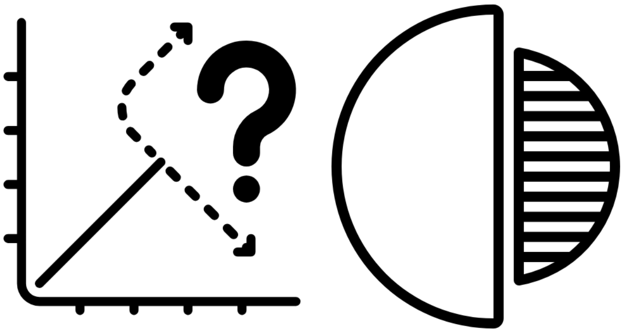

Discussion (1):
The left-middle of the brain

- We think the **left-middle** of the **brain** is **important** for **talking independently**

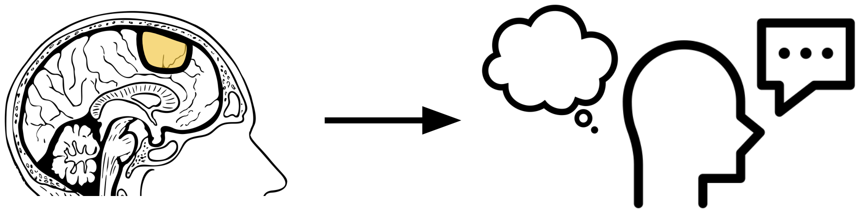

- **Other studies** say this area matters for:
    - Making **choices** about **moving**
    - Doing **difficult tasks**
    - Thinking about **word meanings**
    - Keeping up **energy** during a **long action** ("energization")

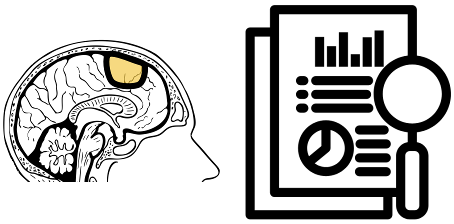

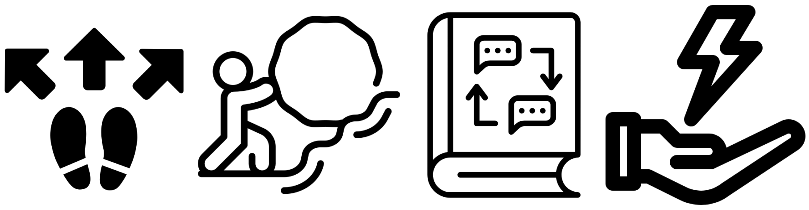

- Some of these ideas **match** well with what we found here

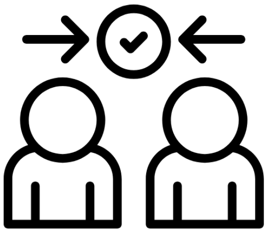

Discussion (2):
Trouble talking independently

- The troubles with talking we saw here are **similar** to other **language problems**, like:

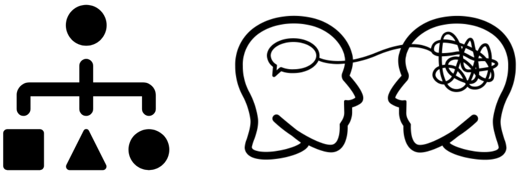

  - “**Transcortical motor aphasia**” (being able to repeat but not talk alone)
  - “**Anomia**” (trouble coming up with words)
  - “**Dynamic aphasia**” (being able to repeat and say common things but not talk alone)

- **Dynamic aphasia** is the **best match** to what we saw here

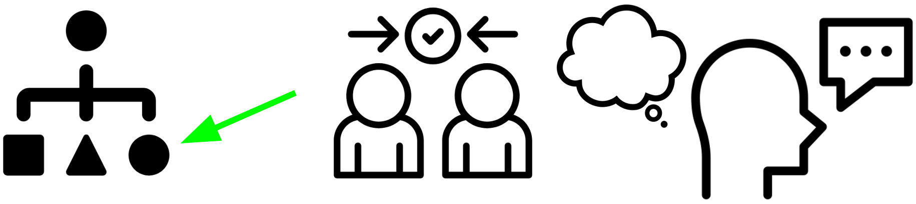

- **Other studies** on **smaller groups** of patients have **also** shown that dynamic aphasia might be related to the middle of the brain

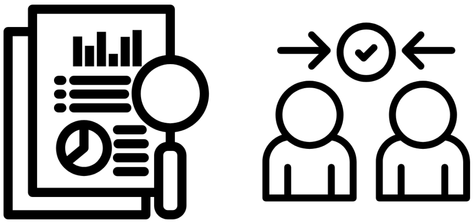

Discussion (3):
Recovery

- **Most** patients in this study could **talk normally** again after about **one month**

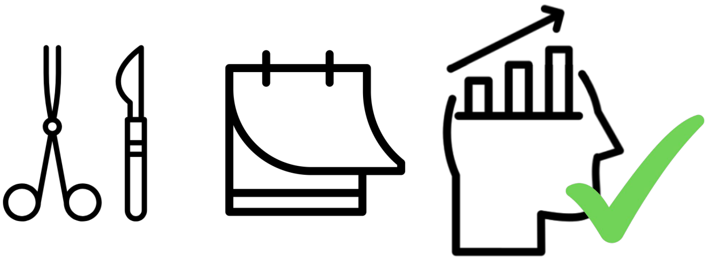

- **However**, this was **not always** the case

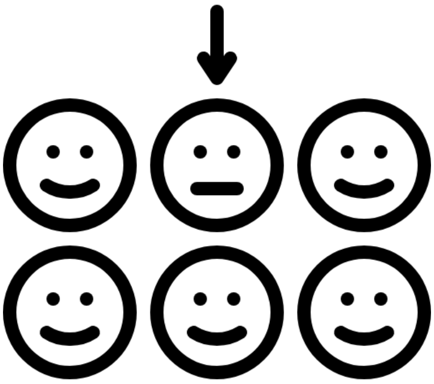

- We want **neurosurgeons** to know to be **careful** around this area

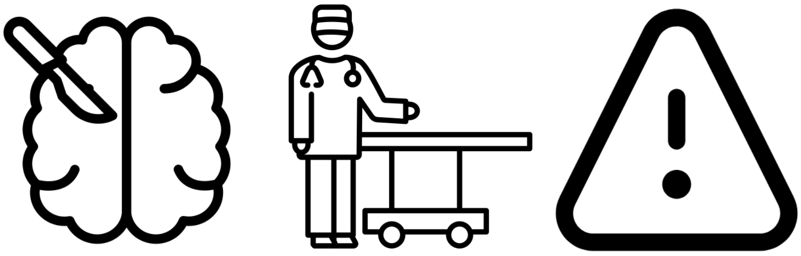

Discussion (4):
Why this matters

- What we found here both **makes sense** with and **expands on** older studies

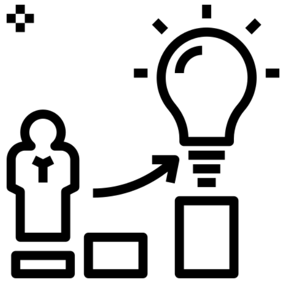

- We think our study is **important** because it helps us **understand** the **brain** and **language**

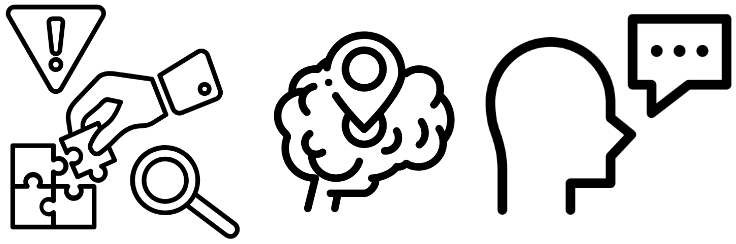

- We **also** think it is **important** because it could help **improve brain surgeries**

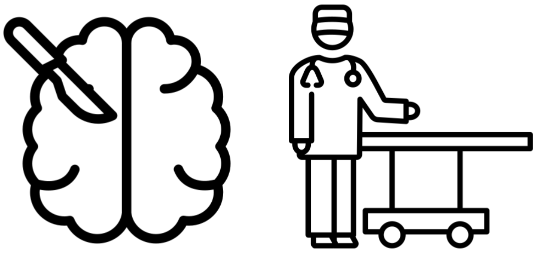

Discussion (5):
Limitations / things to think about

- **Brains** that **need surgery** might be **different** from brains that don't need surgery

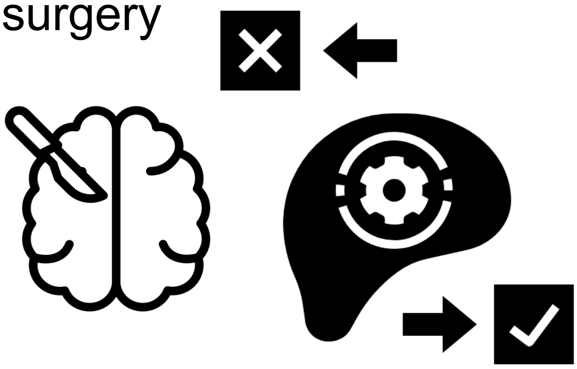

- Our **language tests** were **short** and **don't cover everything** there is to know about language and thinking

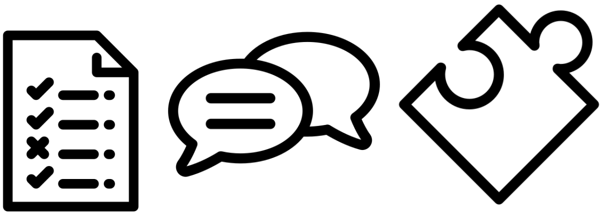

- There is still **a lot to learn** about what **this area** does when it is not damaged

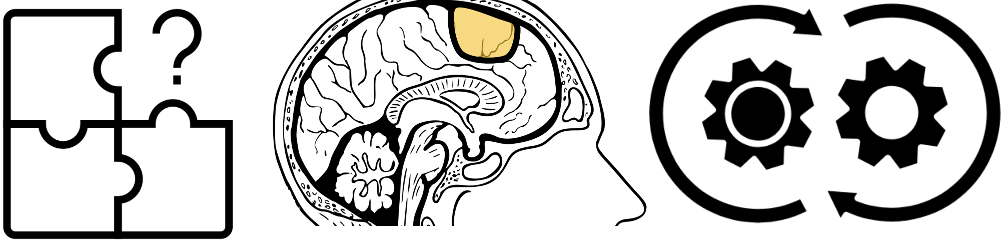

Conclusion

- This study shows that the **middle** of the **brain** on the **left** is related to **talking independently**

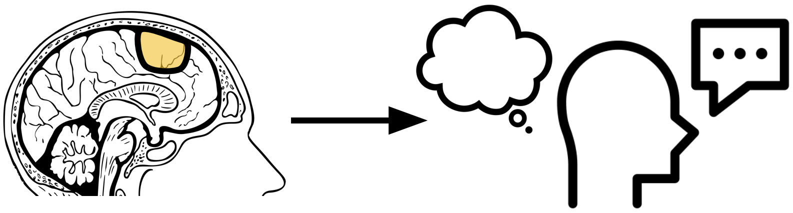

- We think this information is **important** for **understanding language** both in **science** and in **medicine**

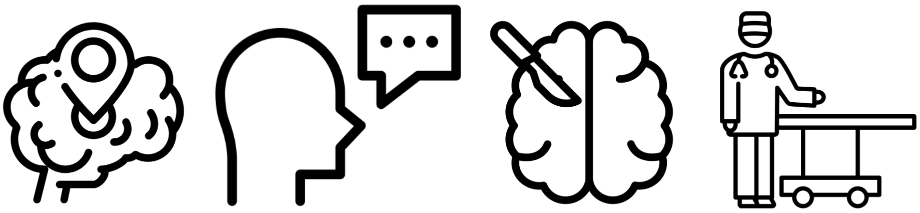

- We hope to **keep studying** this area’s role in language, and hope others will, too!

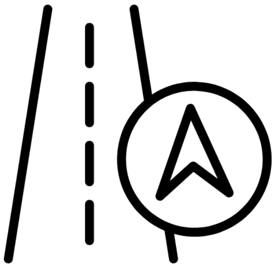

Supplement: Supplementary file 1 [file nol-6-1-166-s001.pdf]
