# Supplementary material for: Role for Left Dorsomedial Prefrontal Cortex in Self-Generated, but not Externally Cued, Language Production
Source: Neurobiol Lang (Camb). 2025 Jun 12;6:nol_a_00166. doi: 10.1162/nol_a_00166 (PMC12170450; doi:10.1162/nol_a_00166)
Supplement: Supplementary file 2 [file nol-6-1-166-s002.pdf]

On April 3, 2023, I had my first ever—and so far only—seizure over Zoom with my then PhD advisor, Andrew Garrett. I had just been offered a position at Yale University and was working toward finishing my thesis. According to Andrew, who had called the emergency services, my initial onset was first characterized by a bout of aphasia. My right arm then suddenly jerked upwards before I fell out of my chair, collapsing onto the wooden floor. After completing a CT scan, the hospital staff informed me that they had found a mass in my brain.

Today, I am a Lecturer at Yale in the Department of Linguistics. I recently graduated from the University of California, Berkeley with a PhD in Linguistics. In addition to historical linguistics, my primary research area involves conducting fieldwork on Indigenous languages of North America, specifically on Northern Pomo, Unangam Tunuu (or Aleut), and Crow. As some people know, Indigenous communities have among the highest rates of poverty, suicide, substance abuse, and among other health-related issues. Through my personal experiences working with and for Indigenous community members, I have grown to become more aware of my privileges in relation to others who may be experiencing their own hardships in life. I also consider myself a realist—others may call me a pessimist—and I mention this because I tend to expect the worst; this has been my outlook since entering academia. *Oh, I got rejected from this fellowship? Well, I expected that!* Going into the surgery and knowing that there was a possibility that I would experience symptoms of aphasia or not be able to walk perhaps permanently, I was already preparing for the worst.

In preparation for my tumor resection surgery, I had several email exchanges and phone conversations with Susanne Gahl, a psycholinguist at UC Berkeley. Among other topics, Susanne specializes in researching aphasia and has personally interacted with individuals experiencing aphasia with symptoms across a wide spectrum. What she highly recommended was designing a visual communication aid so that I could point in the event I had difficulties retrieving words, forming sentences, and the like. I also included audio of myself saying the various prompts and requests on my iPad. For example, there were prompts that included the following, “My name is Edwin! What’s your name?”, “It’s nice to meet you. How are you?”, “Do you have any pets? I have two cats”, and “This is my cat, Turnip. This is my cat, Turtle. Look how cute they are!!” I shared my visual communication aid with Susanne via email.

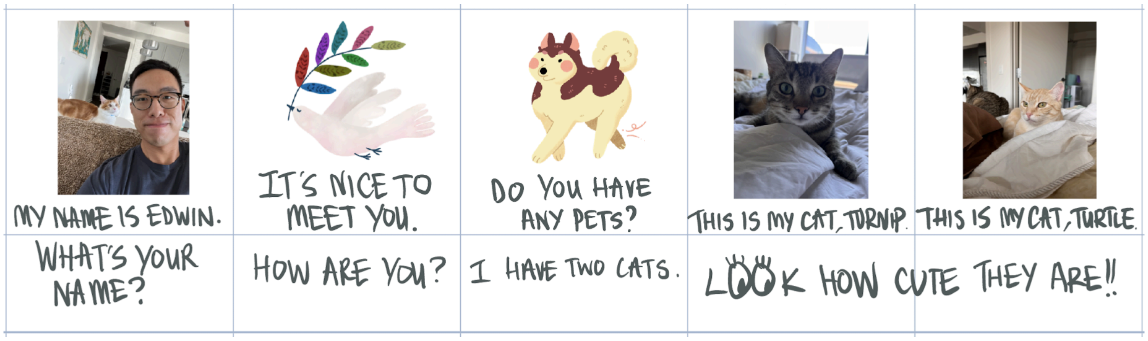

Figure: A single row of my aphasia communication aid.

Susanne responds with the following (p.c., April 22, 2023): “Wow, this is great—and delightful! Turtle and Turnip are indeed *extremely* cute. I also love that you include conversation starters. Aphasia can be so isolating, partly because people without aphasia sometimes assume that people for whom using language is difficult don't want to have any conversations. The National Aphasia Association has a bunch of resources, including descriptions of potentially useful apps. They're much less delightful, but you could take a look: https://www.aphasia.org/helpful-materials/. While I'm at it: The Aphasia Center of California is at https://aphasiacenter.net/. Like I said [on the phone], it's mostly geared towards people with chronic aphasia. But I wish everyone would read the information, regardless: It is *much* easier to learn about aphasia before one has had a stroke or other injury to the brain than afterwards. One other thought: You might print out (and laminate, for more heft) one version of your communication tool that only has very few items, in case motor area syndrome makes it difficult to point with precision.”

In a follow-up email later that day, she writes, “An item just occurred to me that could be really useful to have: ‘Wait, there is something I want to say.’ I don't know [why] it has taken me years to come up with it, or why I haven't seen it before, but I have seen countless situations where it would have been useful. If I could only have three items on my communication board, I think I'd pick that one, plus the scale from negative to positive (which does double-duty as no/yes, less/more and so many other things), plus ‘thank you’ or some other expression of love. Number 4 would be expressions of frustration or sadness or a request to be left alone for a bit. ("Sadness" has a potential drawback: I have never met anyone who *wanted* to have aphasia, temporarily or not. But I have also never met anybody with aphasia who wanted others to feel sorry for them; sympathy can be the most irritating of reactions.)”

***

My resection surgery took place in the early morning on Wednesday, April 26th, 2023. As soon as I awoke after the surgery, I vividly remember nurses asking me standard questions to gauge my general awareness and prompting me to perform some physical activity presumably to assess my physical capabilities. Some of these questions were along the lines of “Where are you?”, “What is the date today?”, “When is your birthday?”, and so on. They would probably ask these questions every five, six hours or so, and if I was asked to rate myself on how I responded, I would give myself a solid A++. It was the next day when Dr. Oberheim Bush, my neuro-oncologist, came to visit that I realized I was experiencing symptoms of aphasia—specifically, difficulties accessing and retrieving words. At first, she assumed I was a software developer. When I informed her I was a linguistics graduate student, she asked me to describe what a linguist does. As I tried to respond to her question, which seemed like a relatively straightforward and fair question to ask, I found myself struggling to express my thoughts in words. I thought to myself, *Hmm. Well, that’s odd. Why do I have difficulty describing what I find so much joy doing?* As I struggled to string words to produce well-formed sentences, Dr. Oberheim Bush informed me that it was typical for people who underwent resection to experience “word-finding difficulties.” Another thing I noticed was that my comprehension remained fully intact.

In what follows, I present excerpts from individuals who interacted with me between my brain tumor resection surgery and my one-month post-surgery follow-up with Deb Levy. With one or two exceptions, all of these quotes and excerpts come from professionally trained linguists. The prompts used varied across individuals, but they generally were framed to elicit salient observations about my language and speech. The prompts also included one or two sentences on whether they felt comfortable having their responses directly copied into the present document. Regarding the email exchanges with Susanne Gahl presented above, I emailed her for permission retrospectively.

Memory can be such a fickle thing. The sequence of events is a bit fuzzy, but here's what I recall. A day after my surgery, the first to visit were my mother and Aunt Silvia Teng. There may have been a restriction on the number of visitors, so when Andrew Garrett, again my then PhD advisor, and one of my friends, Zach O'Hagan, came by, my mother and aunt stepped out. Both Andrew and Zach are professional linguists; Zach had graduated from the same program in 2020. At that time, I was experiencing a high degree of aphasia. Specifically, I found it challenging to access specific words to form coherent sentences and phrases. As Zach, who still uses a flip phone, puts it via text (p.c. December 7, 2023), "You looked heavily lost in thought. Lots of 'um…'." I also remember there were flowers sent by the Linguistics Department at Yale University; I remember this because Andrew had made some comment when he saw the card affixed to the flowers.

Regarding my speech, Andrew made two main observations (p.c., December 10, 2023). In an email, he writes, "My memory of visiting you in the hospital is of course first that it was really nice to see you, and that you seemed generally happy and healthy. As far as language, maybe I should say that as far as I know I had never before encountered a person experiencing aphasia (except with you over Zoom when it all started). In any case, there were two aspects of your language use that really struck me in the hospital. One was that it very often seemed that you had an idea to express, or a thing to say, but couldn't find it. It was like inside your head was a large, large room and you were looking for the thing you needed and could not see it, though you knew exactly what it was. We all have the experience (more as we age) that we sometimes cannot locate a word that we know, and on the inside that feels like looking for a book on a bookshelf where I know I'll recognize it when I see it but I can't tell you what it looks like. It felt with you like that was the predominant thing that was going on for you. You were excellent with yes-no questions, but open-ended questions were not guaranteed to get an answer because of this. I think you may have said 'um' a lot to fill in the pauses. The other striking thing was that some vocabulary, which I would have considered very specialized, was accessible just fine. So you were able to say some very specific complicated things about your surgery or your aphasia, as I recall. It felt as if certain pathways or routines were working just fine, even as many or most were closed off. I can't quite remember the details of which things you were verbally facile with, but there definitely were some. Mostly, though, it felt like the conversation was frustrating for you because you couldn't find the words to express the thoughts that you had."

It is perhaps relevant that between the time of my biopsy and my surgery, in addition to reading up on aphasia, I was reading on neurolinguistics, specifically Jonathan Brennan's 2022 short monograph *Language and the Brain*, and watching YouTube videos on brain tumors and brain tumor survivors. In addition, the metaphors of a "large, large room" and "looking for a book on a bookshelf"

for my mental lexicon very much aligned with my own experiences during the period I had aphasia. As I described to a friend and then related to Deb during the one-month follow-up Zoom meeting, it was as if I was a farmer and all the words were buried beneath the soil. I was constantly trying to find that one specific word in the field that contained all these different words. Except I didn't know where that word was. And so I kept on digging and digging, just trying to locate that one word to no avail. I also felt strongly that the words were not in (direct) competition. Rather, it was as though my mental lexicon was some form of *tabula rasa*—a blank slate. In my situation, however, the main difference was that I had access to a small inventory of eclectic words to work with at the outset.

Virtually everyone who visited me at the hospital appeared uncomfortable and acted awkwardly, perhaps due in part to my inability to produce lucid and cohesive speech, even though I was never that articulate to begin with. One exception I noted immediately was that Andrew seemed to be adept at such situations. I vaguely recall him asking Zach if there was any "gossip" from the department that Zach was willing to share. I also seem to recall Zach, who is usually loquacious, being unusually reticent. Therefore, I responded to Andrew's initial email expressing my surprise upon discovering that he had never interacted with a person with aphasia; his interaction that day was astonishingly well-managed. Andrew wrote back, "That's funny. I remember just becoming very aware that it was hard for you to find the words and thinking that we shouldn't burden you with having to look, so [storytelling] would be the appropriate modality."

Another friend who visited me was Raksit Lau. Raksit was also trained as a linguist and graduated from the same program in 2022. He was accompanied by his partner, Wave, and visited sometime after Andrew and Zach, I think. As I recall, Raksit and Wave did not stay as long as Andrew and Zach. Via Facebook Messenger (p.c. December 9, 2023), Raksit noted, "You had difficulty with lexical retrieval but your sentences were fine. You definitely looked like you understood everything we were saying and you didn't seem afraid or sad or anything. But you were super tired and it seemed like you were frustrated about not being able to get words. We [didn't] notice anything about you having an easier time with words related to surgery, etc. tho[ugh]."

In general, I did not have any difficulty walking. I also did not end up using the communication aid because my aphasia was not considered as chronic so as to require its use. Still, I was glad I created one—you just never know. My jaw hurt a little and I looked (and felt) like a hot mess, but I don't suspect those ailments had any direct influences on my aphasia. I was originally scheduled to be discharged on Saturday, but the hospital staff determined I was well enough to be discharged a day earlier. *Great*, I thought, *no more nurses waking me up in the middle of the night asking me those routine questions*. Sometime in the late morning, I think shortly after Deb Levy conducted the aphasia battery and brief interactions with the physical and occupational therapists, I left the hospital.

During the first few days out of the hospital, I walked around outside for maybe no more than twenty minutes. First, I was accompanied by my brother, Lawrence. Then, when he left to go back to Hong Kong, I would be accompanied by my mother. What I lacked in any actual life skills, I made up for in my knowledge of linguistics and the culture of academia. The latter two were not particularly helpful when trying to get my prescription for senna at the pharmacy inside Walgreens. This was perhaps the first time in a very long time I felt ashamed, not because I was trying to request

medication for something that did not actually require a prescription or that what I was requesting was a stool softener, but because my inability to produce fluent speech was holding up other people in line. Upon self-reflection, I thought, *Sure, it's an inconvenience to other people, but I just received brain surgery! Just look at the staples in my head!*

People mention equality and equity, but it's worth distinguishing the two. A student of mine gave me this apt analogy. Everyone differs in their height. When you build a tall fence, some people may be able to see over it, but others may not. Equality is giving each person a stool (i.e. a chair) of the exact same height. Equity is giving each person a stool that allows every individual to see over the fence. In my scenario, as I interpret it, equality is providing the same amount of time for everyone to articulate one's needs. Since I was experiencing lexical access issues, I needed more time than most other people to articulate my own needs—this is equity. And quite frankly, there were two pharmacists stationed at two windows.

Everyday, I went out to get coffee. Through those interactions, I was accustomed to uttering short responses to "What would you like?". For example, one day I might mutter the two-word response, "Latte, please". Perhaps another day, I would respond with "Could I get a latte, please?". Eventually, my mother would feel comfortable allowing me to get coffee on my own. At that point, I would also get small treats. Once she found out I purchased something that she thought was sweet, which was almost always the case, I would make some excuse, such as 都唔甜*,* 你試吓啦*! dou*^{1} *m*^{4} *tim*^{4}*, lei*^{3} *si*^{3} *haa*^{5} *laa*^{1} "It's not even sweet—you try it!". Thankfully, when she did accept the request, it actually wasn't sweet, just supremely buttery. In sum, I built speech habits around my daily routines, slowly and surely expanding my linguistic repertoire to include novel words or phrases.

For several days, I was just in bed reading or playing video games—specifically, *The Legend of Zelda: Tears of the Kingdom*. The next book I read was Giosuè Baggio's 2022 approximately 220-page volume entitled *Neurolinguistics*, which I finished in a single day, to my mother's chagrin who constantly insisted, 你要唞吓你個腦㗎*! lei*^{3} *jiu*^{3} *tau*^{2} *haa*^{5} *lei*^{3} *go*^{3} *lou*^{5} *gaa*^{3} "You need to rest your brain!". Despite these admonitions, I kept on reading. The third book was the second edition of *Cognitive Neuroscience of Language* by David Kemmerer published in 2023, which I never finished. Nevertheless, while reading the pages on aphasia, I remember seeing all these diagrams of the brain and decision trees relating to an assortment of aphasia diagnoses (see, for example, Kemmer 2020:91, Figure 3.10). Memory of these various figures proved helpful when I was teaching modules on aphasia and neurolinguistics in my *Quantitative Linguistics* class about half a year later.

It was just innocent curiosity that I was reading up on aphasia and neurolinguistics. I thought to myself, *I'll let the trained experts diagnose me*, although I did latch onto the term *anomia*, which I suggested to Deb in the follow-up Zoom meeting. In addition to reading and playing video games, I was also working on my dissertation and attempting to continue what I had initially planned. Typing was not particularly as straightforward as I had hoped. Under normal circumstances, it would have taken much mental acuity and effort to retrieve words, form coherent, well-formed sentences, sequence those sentences in a logical and orderly fashion, etc. With aphasia, it was a struggle to accomplish the same tasks, so much so that all I could manage was click a few buttons on some software then wait for the analysis to run its course, which usually took a couple of hours. During that

time, I would just continue reading or playing video games. As soon as the analyses were completed, I produced graphs and figures and typed in brief captions of what each represented—and even typing the short descriptions for each plot took work.

On the Sunday following my surgery, I was visited by two friends, Julianne Kepner and Tyler Lemon. Both are linguistics graduate students at UC Berkeley, who came bearing gifts from the department—two cards and flowers. Tyler's recollection is provided in the following excerpt (p.c., December 7, 2023, emphasis mine): "I remember that your morphology and syntax were more or less fully intact. Every sentence you spoke was grammatical. However, you were having significant issues with lexical retrieval, and this often slowed you down when you were talking. You seemed to understand everything we were saying to you perfectly, but responses were hard for you. Interestingly, I remember that you were doing a good job retrieving vocabulary related to speech disorders, surgery, and to language and the brain. I think you had been reading up on these things before your surgery, so the concepts were well activated in your brain. In terms of temperament, you were calm, and seemed happy to see us."

Julianne agrees with what Tyler had sent me, but expands on his comment in the following series of texts (p.c. December 7, 2023): "I remember you struggling with lexical retrieval but not with the word ['lexical retrieval']. You had no trouble reading the card we brought you and showed full comprehension of the card and its contents. Having conversations with you was a challenge because of the lexical retrieval, but once a word was activated (that is, once you found it or we reminded you of it) you were able to keep using it during the conversation. I'm trying to come up with a specific example. Maybe it was the word 'flowers'? I think you said something like, 'thank you for the....' And we jumped in with 'flowers?' And you nodded and said 'flowers.' The conversation basically went like that. You never left out function words (it was definitely not telegraphic speech), but needed a word bank provided by us in conversation […] You were easily retrieving words related to psycholinguistics but not more 'basic' vocab."

I found at least two items in the observations presented above particularly striking. First, even though I no longer retained much of the information I had read during that period, people noted my use of technical vocabulary. Second, if the example Julianne offers is representative of what actually happened, then even the mere presence of an object—that is, the flowers that were supposedly from the department but I suspect came from Andrew—was not quite enough to prime me to recall that particular word. Given that I was able to accurately retrieve words associated with pictures shown to me when Deb was conducting the aphasia battery, I was not convinced about the veracity of these observations. In fact, when I followed up with both Tyler and Julianne to assess the verisimilitude of that particular event, they were not willing to stake their lives on it. For example, Tyler remarks (p.c. 13 December, 2023), "That [example] seems quite plausible, but I'm not 100% sure. I remember us handing you flowers and you struggling to put together a response, but I don't remember the specific words in the exchange." Julianne also writes (p.c. 13 December, 2023), "I don't remember for sure. I know it was content words but I'm not sure about flowers specifically. So that was an example of the kind of thing, but I don't remember exact quotes."

By the end of the following Wednesday, I noticed some gradual improvements to my speech. I had less and less difficulty retrieving words. My Aunt Silvia and her daughter, Winnie, live in Union City, CA within the same household. On some occasions, they would come and check up on me. I asked them to comment on aspects of my speech post-surgery. Although my aunt and cousin were not formally trained as a linguist, my cousin's daughter is currently pursuing a PhD in linguistics at the University of Washington. Therefore, by association, my aunt and cousin are basically linguists. Together they offered the following observation (p.c., December 13, 2023): "Three days after surgery, he was quick to answer questions that required only a yes, no, or OK. But for all other questions, requiring more words, he started off with a series of "um… um…" but was unable to find words to finish his reply. Although he was attentive and his facial expression showed immediate comprehension of our questions, I did not try to press him long for conversation. Despite this he did not seem frustrated, probably owing to his understanding about his condition from both personal and scientific perspectives. 11 days [i.e. Sunday, May 7, 2023] after surgery he seemed to return to normal speech, at least as far as light conversational topics went."

Finally, on May 14, 2023, Andrew and I met up for coffee in Oakland, CA, and he recounted the following in the same email exchange presented above (p.c., December 10, 2023): "I have a less clear sense of your language from when we had coffee. It was way better, certainly, different from the hospital; but I think I remember that there were hesitations or pauses at times as you seemed uncertain. You did not seem quite the same verbally as before the surgery. We didn't spend too long together, maybe half an hour or 45 minutes at most; you said that you got tired very quickly and that we should call it a day. Again, of course, I also recall that it was very nice to see you."

It seems that people who had interacted with me on multiple occasions noted gradual improvements to my ability to produce fluent, continuous speech. I was still experiencing some degree of aphasia over two weeks after my tumor resection surgery on May 14, 2023, when I had coffee with Andrew. When I met the radiologist team for the first time, my speech was almost, but not quite, back to its baseline. Reviewing the visit notes, the resident physician, Dr. Lisa Ni, on May 16, 2023 wrote "still has some aphasia, with some word finding difficulty." During that meeting with Dr. Ni, I remember explicitly saying I was still experiencing aphasia even though I may not have come across as such. When I met with Dr. Steve Braunstein, he had remarked that I seemed to have concealed my speech disfluencies well perhaps on account of my being a linguist. By May 24, 2023 when I had a Zoom meeting with Deb, the symptoms had all but dissipated.

As a person experiencing symptoms of aphasia that particularly affected lexical retrieval, yes-no, polar questions were among the most facile. Perhaps most challenging were open-ended questions; it did not ever occur to me how often open-ended questions one encounters in the hustle and bustle of everyday life. I listened attentively whenever my friends and colleagues would engage in lengthy storytelling, especially gossip. I appreciated when my interlocutors stood by patiently as I attempted to re-locate lost words, but I especially appreciated when people would provide their assistance, such as supplying a "word bank"—or a set of words that might match the context. Finally, as Susanne best puts it, "sympathy can be the most irritating of reactions." People mean well, but it can get frustrating when you are constantly treated as a victim. Ultimately, my suggestion is to focus

on things you can control, get into the daily routine of positive affirmations if you haven't already done so, and find and prioritize things that bring value and meaning in your life, which can shift over time. Today, I am thriving and I want to express my deepest gratitude to Dr. Eddie Chang and his team for prolonging what I consider to be the greatest gift of all—life.
