# Supplementary material for: Role for Left Dorsomedial Prefrontal Cortex in Self-Generated, but not Externally Cued, Language Production
Source: Neurobiol Lang (Camb). 2025 Jun 12;6:nol_a_00166. doi: 10.1162/nol_a_00166 (PMC12170450; doi:10.1162/nol_a_00166)
Supplement: Supplementary file 3 [file nol-6-1-166-s003.pdf]

Supplemental Materials

Sample connected speech transcripts from the nine patients who presented with the disproportionate self-generated speech deficit following resection in the lesion-symptom-mapping-based region of interest (LSM-ROI; Fig. 1A). Language was evaluated at 2–3 days post-op unless otherwise noted. Prompts from the examiner and/or event descriptions are displayed in brackets. In cases where output from conversation prompts was limited, picture description transcripts were included where available. Images are the first sagittal acquired T1 images available after the immediate post-op timepoint (time post-op range: 1 month–2 years).

Sample case 1 (as described in "Representative case description")

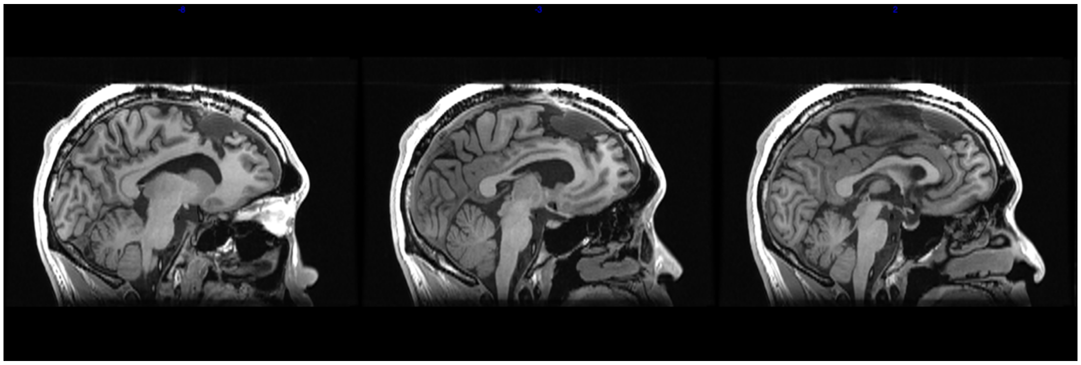

Overall: 8.43

Repetition: 9.17

Confrontation naming: 9.58

Self-generated speech: 5

Connected speech excerpt:

[can you tell me what you remember from the surgery?]

yeah, um...

so I...woke up, um...

I...

um...

I don't know which order this is, but...um...

I...

had a...

picture association (.) [clears throat] task?

um...

and then I...

um...

had a...

um...

...

[sighs]

um...

um...

[are you remembering something and it's hard to get the words for it?]

yeah. [laughs]

[how does it feel to talk right now?]

it feels a little awkward.

...

uh...

I...

but really it has to do with the fact that, um...

I can't (.) get the words out of my mouth?

um...

than...

I am...

hmm.

tf- tf- trying to.

Sample case 2

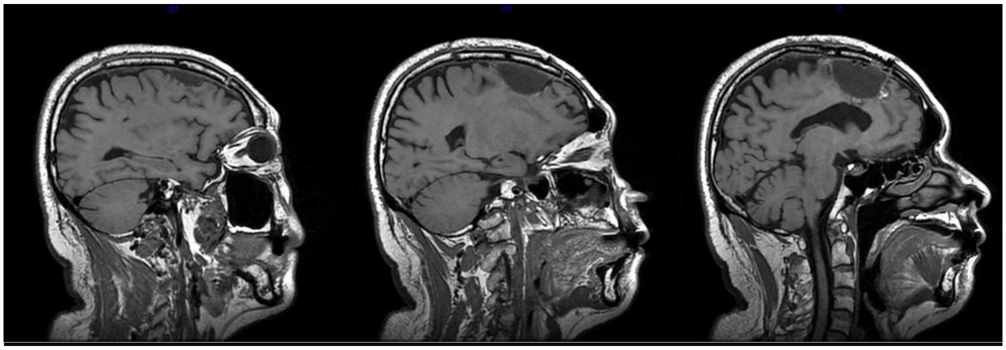

Overall: 4.07

Repetition: 7.1

Self-generated speech: 5.83

Fluency: 1

Connected speech excerpt:

[can you tell me, have you been here before?]

yes.

[can you tell me your full name?]

<right> right at the moment (.) it's uh (...)

[nurse: I know he just walked with (name redacted) and that he said he said he had a little bit of pain.]

[dtr: yes, a little in his back.]

[can you try telling me your name again?]

<it's uh.> [grunt] almost that time.

[what's your address?]

address is (.) figuring out (..) uh.

[what did you do for work before you retired?]

uh (...)

Sample case 3

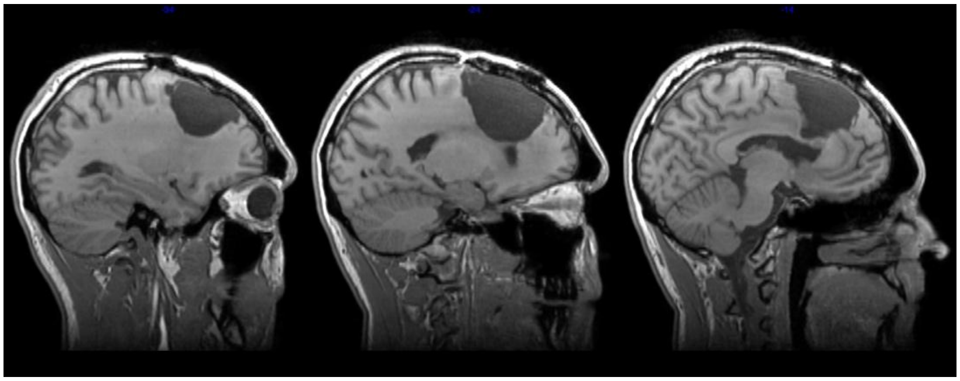

Overall: 5.36

Repetition: 9.1

Confrontation naming: 4.83

Self-generated speech: 2

Connected speech excerpt:

[go ahead and tell me your name.]

um [name redacted].

[good, can you give me your full name?]

um (.) [full name accurately produced]

[and how about your address?]

um (...)

[do you know what street you live on? is that hard to come up with?]

yes but um (...)

[how about the city?]

um (...)

[tell me about the job you were at before.]

yeah um uh (...)

[you were teaching right?]

yeah but um (...) uh (...)

Picture description:

a tree.

light um uh (...)

[anything else?]

(...)

[what’s happening here, in this part of the picture?]

(...)

[do you know what they’re doing?]

yeah uh (...)

Sample case 4

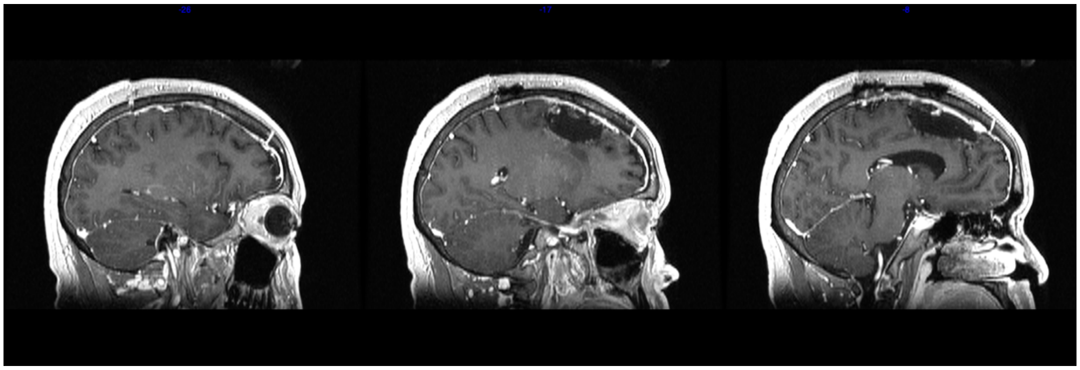

Overall: 6.7

Repetition: 9.8

Confrontation naming: 9.5

Self-generated speech: 2

Connected speech excerpt:

[can you tell me about a job that you either have now or had in the past?]

yeah.

[tell me a little about it.]

<I> I diction.

[and what do you do there?]

um (...) um (...)

[what are some day to day things you do?]

yeah yeah um (...) um (...)

it's on the tip of my tongue.

[do you work with computers?]

yeah.

[and now in a few sentences could you tell me about your surgery on Monday, what happened, and why you're here today?]

right um (...) uh

[what was the surgery for?]

(...) hold on.

um (...)

it's on the tip of my tongue.

[yeah it was for a brain tumor right?]

yeah.

um (...)

Picture description:

um (...)

[do you know what's going on on this side of the picture?]

yeah um (...) yeah.

[let's try some individual things. what's this thing here?]

kite.

[and how about this right there?]

um (..) flag.

[and do you know what they're having, these people?]

um (...)

Sample case 5

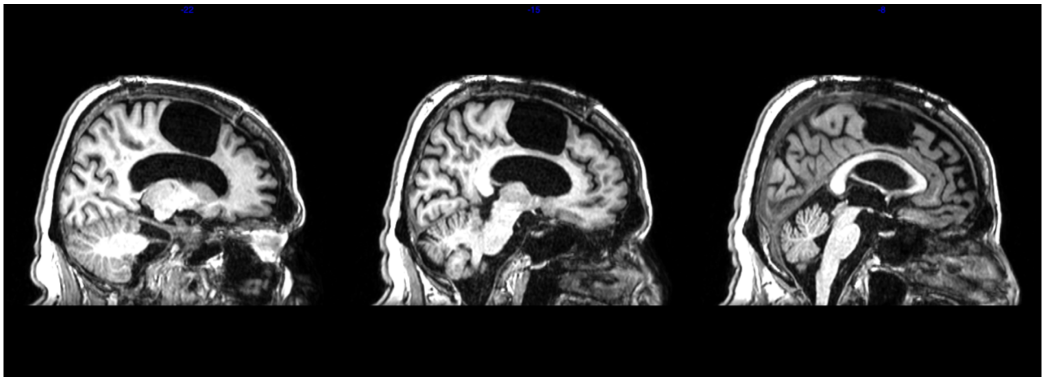

Overall: 6.48

Repetition: 10

Confrontation naming: 4.17

Self-generated speech: 1.67

Connected speech excerpt:

[could you tell me where we are right now?]

mm (..) mm (...)

[can you try and tell me what month is it?]

[no verbal response]

[how old are you?]

[no verbal response]

[are you okay to keep going?]

yeah.

Continued deficit at one month follow-up

Overall: 8.19

Repetition: 9.58

Confrontation naming: 8.33

Self-generated speech: 3.33

[when you woke up from surgery, did you have trouble talking immediately?]

oh, yeah.

oh, yeah.

um…

I (..) had trouble (.) [nods] talking (…) um, immediately.

and… [sighs]

yeah.

[can you tell me about your job a little bit? what you do professionally?]

I am a [job redacted], uh, and a [job redacted].

[oh, wow.]

um…

(…)

that's (…) [sighs] that's it.

[I know you have more to say.]

[laughs] yeah.

Sample case 6

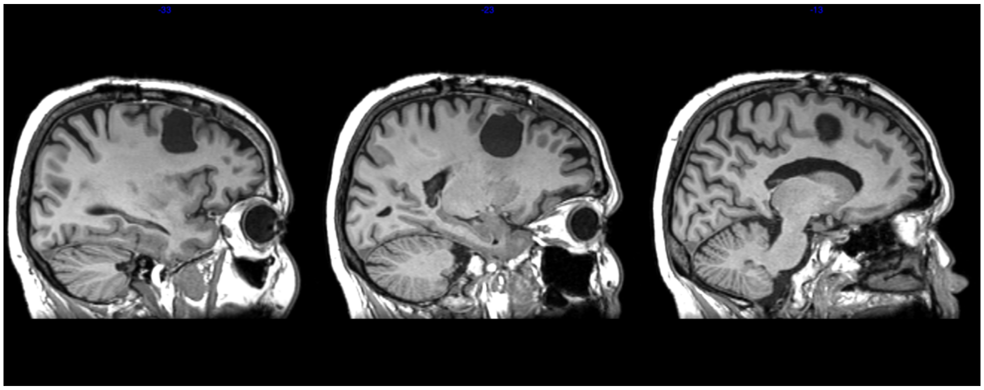

Overall: 9.22

Repetition: 10

Confrontation naming: 8.75

Self-generated speech: 6.67

Connected speech excerpt:

apparently it went well.

so...

I don't know from their...

uh, yeah.

[how's it been in the hospital since then?]

great. all the help has been wonderful.

so...

[you remember those weird sentences?]

yes, definitely.

[how do you think you were doing with the words during the surgery?]

I'd say about, 50/50. [laughs]

Sample case 7

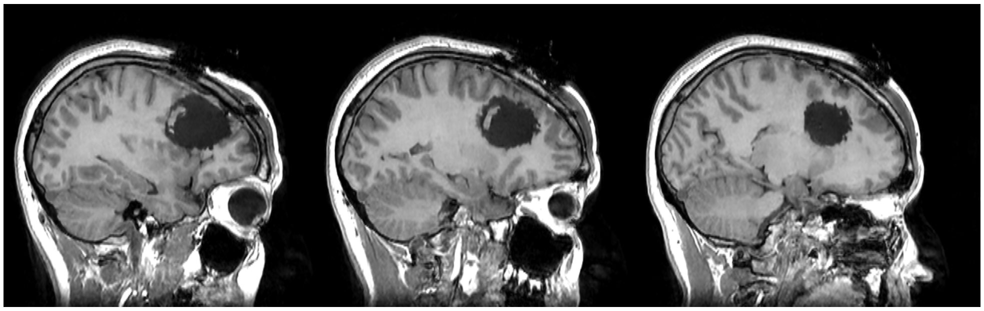

Overall: 7.87

Repetition: 10

Confrontation naming: 7.92

Self-generated speech: 4.17

Connected speech excerpt:

[can you tell me about your kids?]

um I have two kids.

they are um, small.

(…) they (..) uh like to be fʌm.

[they like to fool around?]

they like to be found well.

[how old are they? you said they were small.]

they were five and three.

[you have your hands full.]

yeah.

um (...) uh they were um (...)

they were (...)

[is the three year old in daycare yet?]

yeah.

[so he hasn't started preschool yet?]

no, he's started.

um um (...) study wak

[husband: where is (name redacted) at?]

[name redacted] is at um (...)

[husband: what grade is she in?]

she is in grade (...) three.

[and how old is she?]

<four> five.

Sample case 8

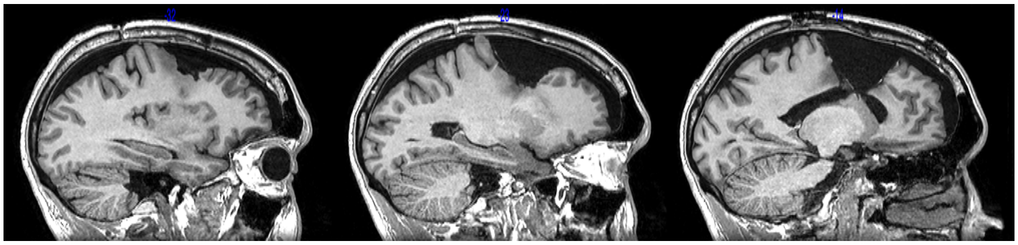

Overall: 4.05

Repetition: 5

Confrontation naming: 6.25

Self-generated speech: 0.83

Connected speech excerpt:

[can you tell me where you're from?]

(...) uh. (...)

[are you from (state redacted)?]

yeah.

[northern (state redacted) or southern (state redacted)?]

yeah um (...)

[are you from close to (city redacted)?]

(..) yeah.

[okay, and you live with your dad right?]

well (...)

[your dad's name is (name redacted), right?]

yeah.

[has he come and visited you so far?]

yeah.

[since the surgery?]

twice.

[can I ask you what your hobbies are? what do you like to do for fun?]

um. (...) yeah.

[do you play any instruments?]

yeah.

[okay, what kind of instruments?]

uh (...)

Sample case 9

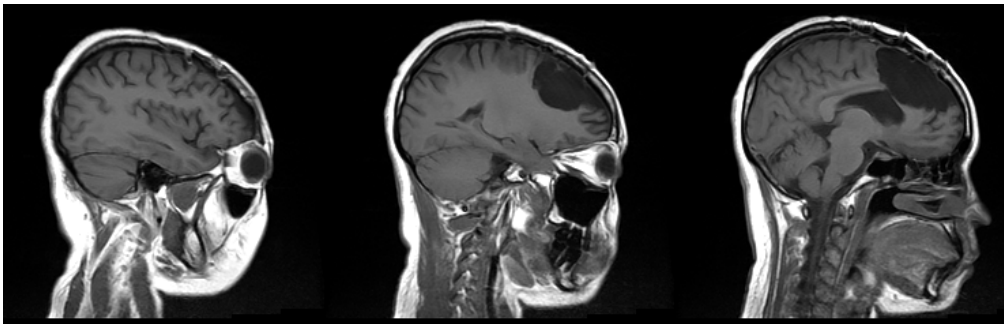

Overall: 9.17

Repetition: 10

Confrontation naming: 9.58

Self-generated speech: 6.67

Connected speech excerpt:

[can you tell me a little bit about what you do for work?]

yeah um (.) I'm a software test engineer.

I (.) could (..) test um (...)

I don't know (.) um, anything.

[what kind of a company do you work at?]

um [company redacted].

[is that a small or big company?]

it's a big company in [city redacted].

um (..) I don't know what they focus on.

I just started in April.

[what were you doing before you started there?]

um (.) I was applying at jobs.
